# A High-Precision Odometry Calibration Method for Mecanum-Wheeled Mobile Robots Based on ZUPT and Closed-Loop Pose Estimation

**DOI:** 10.3390/s26185692

**Published:** 2026-09-08

**Authors:** Tursun Mamat, Longfei Li, Jiake Wuyuncaicike, Chunguang He, Wenliang Zhou, Zhaolong Liu, Qiuju Yang, Li Xu

**Affiliations:** 1 School of Transportation and Logistics Engineering, Xinjiang Agricultural University, Urumqi 830052, China; 320242634@stu.xjau.edu.cn (L.L.); ceceg@xjau.edu.cn (J.W.); hechunguang@xjau.edu.cn (C.H.); zwl@xjau.edu.cn (W.Z.); 320242618@stu.xjau.edu.cn (Z.L.); xuli19940111@163.com (L.X.); 2 Intelligent Transportation Engineering Research Center, Xinjiang Agricultural University, Urumqi 830052, China; 3 Xinjiang Key Laboratory of Transportation and Logistics Engineering, Xinjiang Agricultural University, Urumqi 830052, China; 4 Xinjiang Communications Construction Group Co., Ltd., 840 Wuchang Road Auxiliary Road, Urumqi 830052, China; 15999176681@163.com

**Keywords:** Mecanum-wheeled mobile robot, odometry calibration, closed-loop pose feedback, zero-velocity update (ZUPT), adaptive covariance, intermittent stop-and-go motion

## Abstract

**Highlights:**

**What are the main findings?**

- This study presents a two-level closed-loop calibration framework that combines L2-norm displacement feedback, normalized angular increment integration, relay control with a tolerance deadband, and zero-velocity-based adaptive covariance switching.
- Across the three tested surfaces, the mean absolute longitudinal odometry error was reduced from 53.18 mm to 9.69 mm. For rotational calibration, the Error Reduction Rate ranged from 59.13% to 96.58%.
- The adaptive covariance method was also evaluated with EKF, UKF, RCKF, and a graph-based SLAM optimization framework implemented by slam_toolbox. Across the evaluated back-ends, it reduced the positional RMSE relative to /odom, with reduction rates ranging from 19.38% to 67.44%.

**What are the implications of the main findings?**

- By correcting scale errors during motion and limiting pose drift when the robot is stationary, the proposed method improves the quality of the odometry input used by different pose estimation back-ends.
- It does not require an additional zero-velocity pseudo-measurement node and allows the calibration parameters to be updated online through ROS 2. These features make the framework suitable for stop-and-go warehouse transport and mobile manipulation tasks performed on approximately level surfaces.

**Abstract:**

A two-level closed-loop calibration framework is proposed to reduce odometry scale errors during motion and pose drift during stationary periods in Mecanum-wheeled mobile robots. At the upper calibration level, the planar displacement between the initial and final poses is calculated using the L2-norm, which reduces the influence of lateral deviation on distance measurements based on a single coordinate axis. Rotational displacement is obtained by accumulating normalized angular increments, thereby avoiding discontinuities when the yaw angle crosses the ±π boundary. A relay controller with a tolerance deadband is also introduced to reduce static-friction-induced stalling and oscillation near the target during low-speed calibration. At the lower odometry interface, the covariance assigned to wheel odometry measurements is adjusted according to the commanded zero-velocity state. During stationary periods, this adjustment increases the contribution of near-zero velocity measurements and limits the effect of residual velocity estimates and sensor noise on the fused pose. The identified longitudinal and rotational compensation factors are then updated online in the dead-reckoning node through an ROS 2 service. Unlike conventional ZUPT implementations, the proposed method does not require an additional zero-velocity pseudo-measurement node. Experiments were conducted on three near-horizontal surfaces: ceramic tile, epoxy resin, and asphalt. Across 720 bidirectional in-place rotation trials, the angular Error Reduction Rate ranged from (59.13%) to (96.58%). In 540 straight-line trials covering nine combinations of surface type and target distance, the overall mean absolute error decreased from 53.22 mm before calibration to 9.69 mm after calibration. Intermittent stop-and-go experiments were further performed using the EKF, UKF, RCKF, and a graph-based SLAM optimization framework implemented by slam_toolbox. For each estimation back-end, the estimated trajectory was evaluated by calculating its deviation from the corresponding synchronized /odom trajectory under the fixed-covariance and proposed ZUPT-based adaptive-covariance configurations; /odom was used as a common comparison baseline rather than as an absolute localization ground truth. The adaptive covariance strategy reduced the positional RMSE by (19.38%–67.44%) across the evaluated filtering back-ends. These results show that the proposed framework can reduce both motion-dependent odometry scale errors and stationary pose drift under the tested surface conditions.

## 1. Introduction

In modern intelligent logistics and warehousing systems, automated guided vehicles (AGVs) are increasingly deployed to operate collaboratively with storage infrastructure, robotic workstations, manipulators, and autonomous charging stations. Due to their omnidirectional mobility and superior maneuverability, Mecanum-wheeled robot have seen widespread adoption in this domain [1]. To fulfill the requirements of dynamic, autonomous, and safe operation [2], robust navigation systems are strictly required. Within the autonomous navigation stack, low-level dead reckoning serves as a fundamental component: it provides the primary feedback for control loops while establishing the foundation for high-level multi-sensor fusion localization and global path planning [3]. Consequently, achieving high-fidelity, low-drift odometry remains a critical prerequisite for the advancement of Mecanum-wheeled robot technology.

Odometry accuracy in Mecanum-wheeled robot is largely limited by both the physical characteristics of the system and the inherent constraints of the estimation algorithms. Mechanically, Mecanum wheels utilize discrete rollers angled at 45° for ground contact. This inherent polygonal effect induces high-frequency micro-slip, resulting in strong coupling between the vehicle’s longitudinal and lateral kinematics. Consequently, deviations in the actual rolling radius and kinematic track width from their nominal parameters introduce systematic scale drift under purely open-loop temporal integration [4]. Algorithmically, standard multi-sensor fusion frameworks are limited by the transmission of fixed covariance matrices from low-level drivers to the fusion backend. Even when the robot is stationary, encoder quantization, motor dead-zone effects, and small residual movements may still produce non-zero odometry readings. With a fixed covariance matrix, the filter cannot adjust the measurement weights to reflect changes in the robot’s motion state. Consequently, these disturbances may be introduced into the fusion process and cause fluctuations in the estimated stationary pose.

During low-speed maneuvers, wheel–ground static friction and motor deadband effects reduce the control accuracy of traditional linear controllers [5]. Mechanical servo systems frequently suffer from transmission lags and power efficiency losses due to these nonlinearities, necessitating adaptive robust compensation schemes that address both friction and deadzone simultaneously to achieve precise output positioning. Furthermore, the discontinuous nature of Coulomb friction often causes steady-state oscillations and stiction at motion reversals [6]. Relay-based feedback control strategies have been proven effective in stabilizing mechanical systems afflicted by Coulomb friction. By optimizing the relay gain against the upper bounds of friction disturbances, such nonlinear control laws can enforce finite-time convergence and suppress the limit cycles typically induced by static friction.

Accurate kinematic modeling is fundamental for the control and localization of wheeled mobile robots. However, traditional modeling approaches are often fragmented and tailored to specific mechanical layouts, highlighting the need for comprehensive frameworks that can describe heterogeneous architectures, including omnidirectional systems [7]. The motion of a Mecanum-wheeled robot is determined by the rotational speeds and directions of its four independent wheels. To simplify the kinematic analysis of the chassis, ref. [8] proposed a vector-based modeling method. In practical applications, odometry accuracy is also affected by inconsistencies in actuator parameters. Instead of using a unified velocity model for the entire chassis, ref. [9] established independent speed models for each motor to reduce translational and rotational errors. To further improve parameter estimation accuracy, ref. [10] introduced an Unscented Kalman Filter (UKF) for the joint estimation of kinematic parameters and sensor extrinsics. This method enables online parameter updates during robot operation and improves the accuracy of odometry and pose estimation. Previous studies have shown that errors in wheel radius, track width, and actuator parameters can be corrected through model identification. However, the wheel–ground slip associated with Mecanum wheels varies with the surface condition. As a result, calibration parameters obtained from nominal geometric models or tests conducted under a single motion condition may not remain valid across different types of terrain.

Neural networks have been used to compensate for systematic odometry errors by learning nonlinear relationships between motion states and accumulated drift from historical data. However, their performance is closely tied to the conditions represented in the training dataset. Changes in terrain, payload, robot configuration, or operating conditions may therefore reduce their accuracy when the model is applied to previously unseen scenarios. By comparison, online calibration methods based on kinematic constraints retain a clear physical interpretation and do not require large-scale training datasets. This makes them more suitable for real-time correction when the robot operates under changing conditions. Furthermore, various investigators have demonstrated that during closed-loop autonomous calibration, traditional PID controllers exhibit fundamental limitations when addressing mechanical constraints. Specifically, as the vehicle approaches the target distance or angle, the PID control output asymptotically decays toward zero. This diminished output is frequently insufficient to overcome static friction, resulting in premature system stalling or the induction of limit-cycle oscillations around the target [11]. System architecture constitutes an additional structural limitation. In most existing frameworks, chassis calibration modules remain tightly coupled to low-level runtime nodes. Consequently, the application of new parameters necessitates system downtime or firmware reflashing, a process that disrupts the continuity between calibration computation and real-time execution [12]. To enhance system stability and robustness, numerous researchers have explored diverse control strategies. For instance, various investigators [13,14,15,16] have developed nonlinear controllers for Mecanum-wheeled robot by formulating motor torque directly as the control input. Furthermore, the widespread adoption of high-precision DC motors has enabled the academic community to derive highly accurate motor-driven kinematic models.

In Kalman filter-based navigation, the effective execution of zero-velocity updates necessitates robust stationary-state detection. Numerous scholars have primarily categorized existing detection methods into three mechanisms: kinematic monitoring (velocity or position), inertial magnitude thresholding combined with generalized likelihood ratio tests, and variance-based analysis of inertial data [17]. Upon confirmation of a stationary state, researchers typically employ two principal correction schemes. The Zero Velocity Update (ZUPT) imposes a strict zero-velocity constraint on the filter’s measurement equation. Concurrently, since the vehicle orientation remains invariant during standstill, a Zero Angular Rate Update (ZARU) is introduced into the heading model. The integration of these constraints effectively suppresses the drift of velocity and heading estimates, which is an essential requirement for single-antenna integrated systems [18,19,20,21].

Extended Kalman Filtering (EKF) is widely used in multi-sensor fusion systems for mobile robot localization. However, fixed covariance configurations often exhibit limited adaptability under changing motion conditions, which may lead to pose drift accumulation. To address this issue, several studies have introduced adaptive covariance adjustment methods. For example, ref. [22] proposed a switching-based covariance update strategy for online parameter adaptation during robot operation. In addition to covariance optimization, Zero-Velocity Update (ZUPT) has also been applied to drift suppression in inertial navigation and robotic localization. Previous studies [23] showed that ZUPT can reduce pose drift during stationary periods by constraining the accumulation of IMU errors. This characteristic is particularly important for Mecanum-wheeled platforms, where wheel slip and lateral motion effects often reduce the accuracy of conventional odometry estimation.

Estimation back-ends such as the EKF, UKF, robust cubature Kalman filter (RCKF), factor graph optimization (FGO), and data-driven models differ in how they propagate nonlinear states, handle abnormal measurements, and use historical information. However, the accuracy of pose estimation still depends strongly on the quality of the input observations. When wheel-radius errors, track-width deviations, or wheel–ground slip introduce systematic scale errors, replacing the back-end estimator alone cannot remove these biases. They must instead be identified and compensated for in the motion or measurement model.

Previous studies have investigated kinematic parameter calibration, low-speed control, and zero-velocity-aided localization, but these topics are often treated separately. As a result, there is still a lack of a unified closed-loop calibration framework that combines dynamic scale correction with the suppression of stationary drift. This separation limits the ability of the two mechanisms to work together during actual robot operation.

Several practical difficulties also remain. In linear calibration, a conventional single-axis trajectory may be affected by lateral slip and small heading changes, both of which are common in Mecanum-wheeled platforms. In rotational calibration, large-angle motion can produce discontinuities when the yaw angle crosses the ±π boundary. In addition, many existing experiments have been conducted on a single surface type or with only one fusion back-end. Their applicability across different estimation architectures and under more complicated wheel–ground interactions has therefore not been sufficiently examined.

Accordingly, this study does not aim to develop a general-purpose multi-sensor fusion method or to compare different pose estimation back-ends under standard localization settings. Instead, it focuses on improving the accuracy of Mecanum-wheel odometry during motion and reducing pose drift when the robot is stationary. During motion, closed-loop pose feedback is used to estimate compensation factors for longitudinal and rotational scale errors. During stationary periods, a zero-velocity-based covariance adjustment changes the confidence assigned to wheel odometry measurements and limits the accumulation of noise. Because the proposed method operates at the odometry input level, it can be used with different downstream pose estimation frameworks without requiring changes to their basic structure.

To address these limitations, this study develops a two-level closed-loop calibration framework for Mecanum-wheel odometry. At the upper level, the planar distance between the initial and final poses is calculated using the L2-norm. This reduces the influence of lateral slip and small heading changes that may distort conventional single-axis distance measurements. Rotational motion is evaluated by integrating normalized angular increments, which provides a continuous angle measurement when the yaw crosses the ±π boundary. A relay controller with a tolerance deadband is also introduced to improve stopping stability during low-speed calibration.

At the lower odometry level, the observation covariance is adjusted according to the detected motion state. When the robot is stationary, the covariance assigned to wheel odometry is reduced to limit the effect of small residual velocities and sensor noise on the fused pose. The identified longitudinal and rotational compensation factors are then transmitted to the dead-reckoning node through ROS 2 services. In this way, parameter identification, motion control, and online compensation are integrated into a closed-loop calibration process.

**Figure 1:**
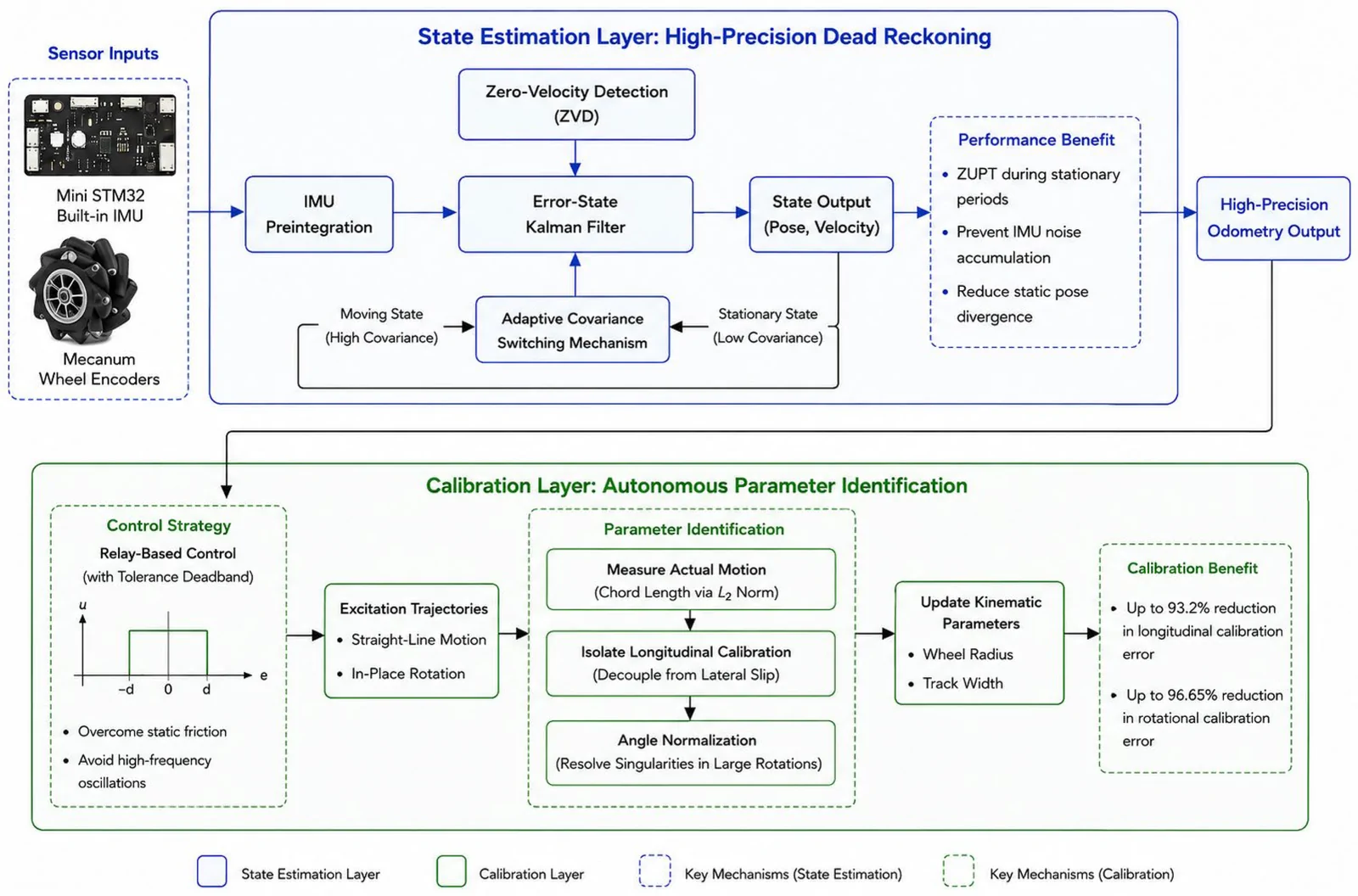
Overall architecture of the proposed two-level closed-loop odometry calibration framework for a Mecanum-wheeled mobile robot, integrating high-precision state estimation with autonomous kinematic parameter identification.

The proposed calibration framework mainly consists of three components: kinematic velocity observation, relay-based motion control, and a zero-velocity-state-based covariance adaptation mechanism (hereafter referred to as ZUPT-based covariance adaptation). At the beginning of the calibration process, the system enters a stationary stage in which the proposed zero-velocity-based covariance adaptation mechanism reduces the odometry covariance to a very small value ($10^{-9}$). This process suppresses pose fluctuations during initialization and provides a stable reference state for subsequent pose estimation. When motion initiates, the covariance is inflated to a large value ($10^{6}$). Meanwhile, the velocity observer utilizes L2-norm and normalization operators to filter Mecanum-wheel lateral slip and Euler-angle singularities, feeding isolated, genuine pose errors to the relay controller. For errors outside the tolerance deadband, a full-amplitude velocity command is executed. Upon entering the deadband, the controller immediately truncates the velocity command. The resulting damping-governed deceleration effectively neutralizes limit-cycle oscillations near the target. Concurrently, this zero-velocity command reactivates ZUPT, returning the covariance to near-zero and preventing error integration from transient braking inertia. Finally, based on steady-state resting pose data, an upper-level module extracts optimal kinematic compensation factors. Utilizing ROS 2 distributed services, these parameters are hot-reloaded into low-level integrators, finalizing a closed-loop architecture that seamlessly couples low-level denoising, mid-level feedback, and high-level autonomous parameter updating. The overall architecture of the proposed two-level closed-loop odometry calibration framework is illustrated in Figure 1.

## 2. Materials and Methods

### 2.1. Kinematic Modeling and Error Source Analysis of a Mecanum-Wheeled Robot

#### 2.1.1. Kinematic Model of the Mecanum-Wheeled Robot

The Mecanum-wheeled robot comprises four independent drivetrain assemblies integrated with a rigid-axle suspension framework.

**Figure 2:**
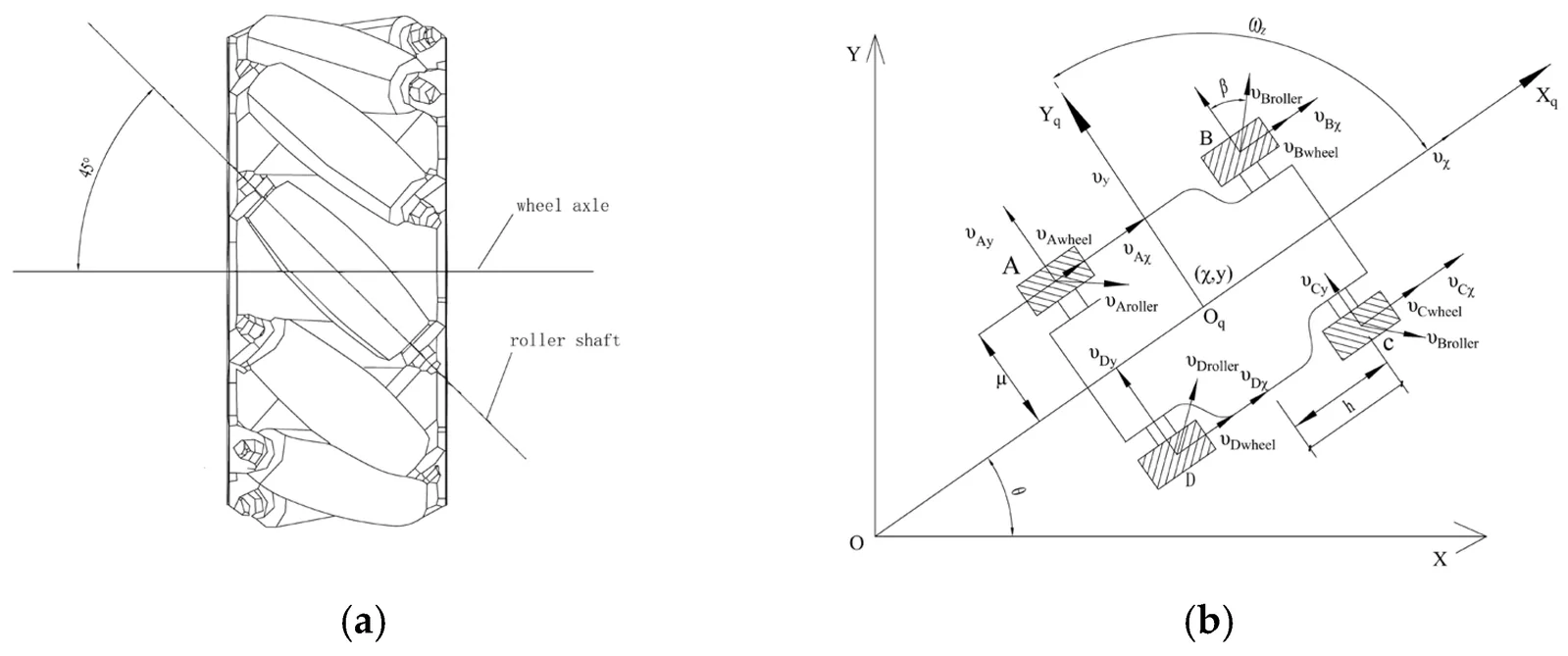
Structure and kinematic modeling of the Mecanum wheel assembly: (**a**) Structural diagram of a single Mecanum wheel; (**b**) kinematic vector analysis of the four-wheel chassis.

**Figure 3:**
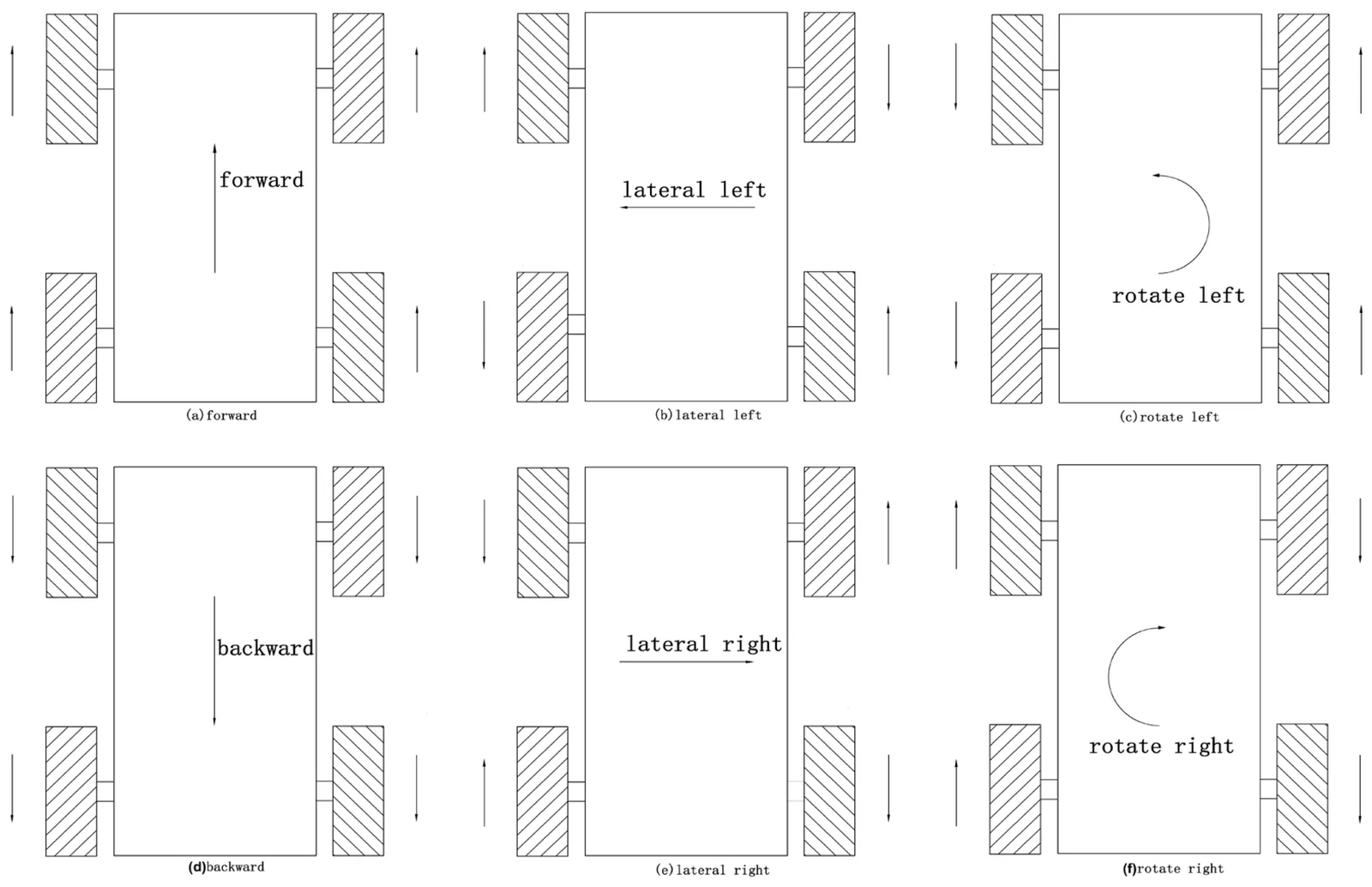
Motion patterns of the four-wheel Mecanum-wheeled robot: (**a**) forward motion; (**b**) leftward translation; (**c**) in-place left rotation; (**d**) backward motion; (**e**) rightward translation; (**f**) in-place right rotation.

Each Mecanum wheel assembly consists of a wheel hub and passive rollers. Due to the unique wheel configuration in which the passive rollers are oriented at a 45° angle relative to the wheel axis (as illustrated in Figure 2a), the robot is capable of executing lateral motion, diagonal motion, and in-place rotation. This characteristic makes the Mecanum-wheeled robot particularly suitable for operation in navigation-constrained and geometrically complex environments [24]. The motion patterns of the four wheels corresponding to forward motion, backward motion, leftward translation, rightward translation, left rotation, right rotation, and in-place rotation are illustrated in Figure 3.

The kinematic analysis of the Mecanum-wheeled robot is derived from the coordinate frames and vector relationships detailed in Figure 2b.

Assuming the robot operates strictly on a planar surface, the kinematic analysis considers only 2D motion. As illustrated in Figure 2b, XOY denotes the global coordinate frame, while $X_{a}O_{a}Y_{a}$ represents the body-fixed frame. Geometrically, the chassis is parameterized by w and h, which denote the lateral and longitudinal half-distances between the wheel centers, respectively. The variable ${\left[x,y,\theta \right]}^{T}$ fully defines the vehicle’s global pose. Additionally, the angle β between the wheel axis and the passive rollers is fixed at 45° for all four wheels. Let ${\left[V_{Ax},V_{Bx},V_{Cx},V_{Dx}\right]}^{T}$ and ${\left[V_{Ay},V_{By},V_{Cy},V_{Dy}\right]}^{T}$ denote the velocity components of each wheel along the $X_{a}$, $Y_{a}$ directions of the body frame. During motion, ${\left[v_{Awheel},v_{Bwheel},v_{Cwheel},v_{Dwheel}\right]}^{T}$ represents the motor-driven tangential velocity, whereas ${\left[v_{Aroller},v_{Broller},v_{Croller},v_{Droller}\right]}^{T}$ indicates the linear velocity of the rollers at the ground contact interface, induced by friction. The variable ${\left[v_{x},v_{y},\omega_{z}\right]}^{T}$ defines the overall body velocity of the Robot. Ultimately, the core objective of this kinematic analysis is to establish the fundamental relationship between ${\left[v_{Awheel},v_{Bwheel},v_{Cwheel},v_{Dwheel}\right]}^{T}$ and ${\left[v_{x},v_{y},\omega_{z}\right]}^{T}$.

Modeled as a rigid body with invariant dimensions, the Robot features a chassis strictly rigidly connected to its four Mecanum wheels. This mechanical coupling ensures that the vehicle body velocity perfectly maps to the individual wheel velocities. By combining equation ${\left[v_{Aroller},v_{Broller},v_{Croller},v_{Droller}\right]}^{T}$ and equation ${\left[v_{Awheel},v_{Bwheel},v_{Cwheel},v_{Dwheel}\right]}^{T}$, the center-of-mass velocities for wheels A, B, C, and D are obtained. Furthermore, the magnitude and direction of ${\left[v_{Aroller},v_{Broller},v_{Croller},v_{Droller}\right]}^{T}$ depend entirely on the differential wheel speeds controlled by the motors and the specific mechanical configuration of the Mecanum wheels. Thus, the center velocity of each wheel can be formulated as follows:

(1) $$\begin{array}{c} v_{Ax}=v_{x}-w\omega_{z}=v_{Awheel}+v_{Aroller}\sin\beta \\ v_{Bx}=v_{x}-w\omega_{z}=v_{Bwheel}+v_{Broller}\sin\beta \\ v_{Cx}=v_{x}+w\omega_{z}=v_{Cwheel}+v_{Croller}\sin\beta \\ v_{Dx}=v_{x}+w\omega_{z}=v_{Dwheel}+v_{Droller}\sin\beta \end{array}$$

(2) $$\begin{array}{c} v_{Ay}=v_{y}-h\omega_{z}=-v_{Aroller}\cos\beta \\ v_{By}=v_{y}+h\omega_{z}=v_{Broller}\cos\beta \\ v_{Cy}=v_{y}+h\omega_{z}=-v_{Croller}\cos\beta \\ v_{Dy}=v_{y}-h\omega_{z}=v_{Droller}\cos\beta \end{array}$$

By computation, the following result was obtained:

(3) $$\left\{\begin{array}{c} v_{Awheel}=v_{x}+v_{y}-(w+h)\omega_{z}\\ v_{Bwheel}=v_{x}-v_{y}-(w+h)\omega_{z}\\ v_{Cwheel}=v_{x}+v_{y}+(w+h)\omega_{z}\\ v_{Dwheel}=v_{x}-v_{y}+(w+h)\omega_{z} \end{array}\right.$$

Let ${\left[\omega_{A},\omega_{B},\omega_{C},\omega_{D}\right]}^{T}$ denote the angular velocities of the four Mecanum wheels and r represent the wheel radius. By applying the velocity transformation from the equation $v_{i}=r\cdot \omega_{i}(i\in \{A,B,C,D\})$, the kinematic mapping between the robot’s body-fixed velocity and the wheel angular velocities is derived as follows:

(4) $$\left[\begin{array}{c} v_{x}\\ v_{y}\\ \omega_{z} \end{array}\right]=\frac{r}{4}\cdot \left[\begin{array}{cccc} 1 & 1 & 1 & 1\\ 1 & -1 & 1 & -1\\ -1/(w+h) & -1/(w+h) & 1/(w+h) & 1/(w+h) \end{array}\right]\left[\begin{array}{c} \omega_{A}\\ \omega_{B}\\ \omega_{C}\\ \omega_{D} \end{array}\right]$$

Based on the geometry in Figure 2b, the velocity transformation between the global and body-fixed coordinate frames is derived as follows:

(5) $$\left[\begin{array}{c} \overset{\cdot }{x}\\ \overset{\cdot }{y}\\ \overset{\cdot }{\theta } \end{array}\right]=\left[\begin{array}{ccc} \cos\theta & -\sin\theta & 0\\ \sin\theta & \cos\theta & 0\\ 0 & 0 & 1 \end{array}\right]\left[\begin{array}{c} v_{x}\\ v_{y}\\ \omega_{z} \end{array}\right]$$

(6) $$\left\{\begin{array}{c} \overset{\cdot }{x}=\frac{r}{4}[(\omega_{A}+\omega_{B}+\omega_{C}+\omega_{D})\cos\theta -(\omega_{A}-\omega_{B}+\omega_{C}-\omega_{D})\sin\theta ]\\ \begin{array}{l} \overset{\cdot }{y}=\frac{r}{4}[(\omega_{A}+\omega_{B}+\omega_{C}+\omega_{D})\sin\theta +(\omega_{A}-\omega_{B}+\omega_{C}-\omega_{D})\cos\theta ]\\ \overset{\cdot }{\theta }\frac{r}{4(w+h)}[(-\omega_{A}-\omega_{B}+\omega_{C}+\omega_{D})] \end{array} \end{array}\right.$$

#### 2.1.2. Dead Reckoning Model and Systematic Proportional Error Analysis

In robot dead reckoning, manufacturing tolerances in wheel diameter, wheelbase assembly errors, and ground slip naturally introduce systematic proportional errors into the wheel odometry [25]. To achieve accurate localization, a linear scaling coefficient is applied to proportionally compensate the raw odometric output. This approach effectively mitigates biases caused by kinematic deviations, thereby enhancing the vehicle’s positioning accuracy during extended straight-line travel.

Within each control cycle Δt, the algorithm receives a 3D body-fixed velocity command from the upper-level planner, as formulated in the equation $v_{t}^{b}={\left[v_{x}\left(t\right),v_{y}\left(t\right),\omega_{z}\left(t\right)\right]}^{T}$. Because of the short duration of this 50 Hz control loop, the Robot’s motion is assumed to be strictly uniform over a single time step.

To obtain the Robot’s absolute global pose as defined in the equation $P_{t}^{n}={\left[x(t),y(t),\theta (t)\right]}^{T}$, we map the body-frame velocity increments to the global frame using the 2D rotation matrix in $R_{b}^{n}(\theta )$. Next, by incorporating the linear and angular velocity calibration factors from $K_{\mathrm{linear}}$ and $K_{angular}$—supplied by the upper-level autonomous calibration algorithm—we construct a discrete-time spatial integration model with feedforward compensation as follows:

(7) $$\left[\begin{array}{c} x(t)\\ y(t) \end{array}\right]=\left[\begin{array}{c} x(t-\Delta t)\\ y(t-\Delta t) \end{array}\right]+\left[\begin{array}{cc} \cos\theta (t-\Delta t) & -\sin\theta (t-\Delta t)\\ \sin\theta (t-\Delta t) & \cos\theta (t-\Delta t) \end{array}\right]\left[\begin{array}{c} v_{x}(t)\cdot \Delta t\\ v_{y}(t)\cdot \Delta t \end{array}\right]$$

(8) $$\theta (t)=\theta (t-\Delta t)+\omega_{z}(t)\cdot \Delta t$$

To meet ROS 2 orientation standards, we convert the estimated yaw angle into its quaternion representation, as shown in the equation $q\left(t\right)={\left[q_{\omega },q_{x},q_{y},q_{z}\right]}^{T}$. Since the robot operates strictly in a planar environment, we assume zero roll and pitch, simplifying the quaternion conversion model as follows:

(9) $$q(t)={\left[\cos(\frac{K_{angular}\cdot \theta (t)}{2}),0,0,\sin(\frac{K_{angular}\cdot \theta (t)}{2})\right]}^{T}$$

Finally, the low-level node publishes a high-precision pose vector after scale compensation $P_{pub}(t)={\left[K_{linear}\cdot x(t),K_{linear}\cdot y(t),q(t)\right]}^{T}$. This architecture directly embeds the calibration parameters into the underlying integration loop, ensuring real-time error correction during the execution phase.

### 2.2. Autonomous Closed-Loop Calibration Strategy for Angular and Linear Velocities of an Omnidirectional Chassis

To identify the longitudinal compensation factor $K_{linear}$ and the rotational compensation factor $K_{angular}$ derived from the physical kinematic model in Section 2, the inaccuracies and inefficiencies inherent in conventional manual open-loop measurements are bypassed. Consequently, an autonomous calibration algorithm driven by closed-loop spatial pose feedback is developed at the upper decision layer. Given that the translational and rotational motions of the Mecanum-wheeled robot are kinematically orthogonal, and that the physical origins of their associated errors (e.g., lateral slip disturbances versus pose singularities) are fundamentally distinct, the calibration process is decoupled into two independent dimensions: longitudinal and rotational.

#### 2.2.1. Discontinuity-Resistant Incremental Angular Velocity Integration Correction

In the autonomous angular-velocity calibration of a Mecanum-wheeled robot, the periodic symmetry of spatial rotation renders conventional absolute angle measurements susceptible to phase wrapping and discontinuous jumps at the ±π yaw angle boundary. When the Robot’s actual rotation crosses this threshold, the sensor-reported angular difference exhibits a spurious step error of 2π, thereby inducing divergence and instability in conventional closed-loop controllers [26]. To resolve this kinematic singularity, an anti-discontinuity nonlinear incremental angle integration model is developed.

First, the algorithm monitors the ROS 2 TF tree at a high frequency to acquire the spatial quaternion $q={\left[q_{w},q_{x},q_{y},q_{z}\right]}^{T}$ of the robot chassis in the world coordinate frame. By exploiting the isomorphic relationship between quaternions and direction cosine matrices, the system then maps this quaternion to the Euler yaw angle $\theta_{raw}$, computed as follows in Equation (10):

(10) $$\theta_{raw}=\arctan(\frac{R_{21}}{R_{11}})=\arctan(\frac{2(q_{\omega }q_{z}+q_{x}q_{y})}{1-2(q_{y}^{2}+q_{z}^{2})})$$

To resolve discontinuities in absolute angle measurements, the direct use of absolute angles is bypassed; instead, the relative rotational increment $\Delta \theta_{raw}$ within adjacent sampling intervals Δt is extracted. To prevent numerical divergence when crossing the ±π boundary, a normalization operator WrapTopi(∙) is introduced. This operator applies a modulo function to the inter-frame difference, thereby mapping the robot’s true angular increment strictly to the interval [−π, π]. Subsequently, an angular-velocity scaling correction factor $K_{angular}$ is incorporated, and discrete incremental integration is performed in the time domain to yield a continuous, singularity-free accumulated rotation angle $\theta_{calib}$, formulated as follows in Equation (11):

(11) $$\theta_{calib}=\theta_{calib}(t-\Delta t)+K_{angular}\cdot WrapTopi(\theta_{raw}(t)-\theta_{raw}(t-\Delta t))$$

Implementing the normalization operator not only mitigates high-frequency step discontinuities at singular crossings but also overcomes the single-turn measurement constraints inherent in conventional algorithms. This formulation permits continuous rotational calibration across arbitrarily large angles, fundamentally attenuating encoder quantization noise and Gaussian stochastic disturbances induced by Mecanum-wheel lateral scrubbing.

#### 2.2.2. Euclidean Geometric Longitudinal Odometry Scale Compensation Based on the L2 Norm

Unlike conventional wheels with continuous rolling contact, Mecanum wheels rely on periodic roller–ground interactions. During motion, the contact point continuously shifts between adjacent rollers, introducing impact disturbances and vibration. These effects can degrade encoder measurement consistency and increase the likelihood of micro-slip during low-speed motion [27]. The latter originates from periodic variations in the effective rolling radius, leading to pulsating deviations in chassis output velocity and causing scale drift between the odometry-estimated displacement and the actual physical displacement. In conventional calibration schemes, the traveled distance is typically inferred by integrating the real-time linear velocity obtained via high-frequency monitoring. However, this high-rate state estimation imposes substantial computational loads on the onboard controller and renders the time-domain integration of velocity noise susceptible to secondary error divergence. To balance engineering applicability and computational efficiency, a proportional correction factor $K_{linear}$ is incorporated into the proposed linear velocity control law. This factor mitigates the nonlinear disturbances induced by the aforementioned physical characteristics on localization accuracy.

The self-calibration of linear velocity is reformulated as a point-to-point displacement tracking problem in two-dimensional space. Initially, the translation vector in the world coordinate frame is denoted as $P_{0}={\left[x_{0},y_{0}\right]}^{T}$. During motion, the real-time translation vector at time t is acquired via TF transformations and denoted as $P_{t}={\left[x_{t},y_{t}\right]}^{T}$.

When executing nominally straight-line commands, Mecanum-wheeled chassis exhibit lateral slip due to uneven inter-wheel friction and IMU installation misalignment. Consequently, estimating the traveled distance via single-axis coordinate differences introduces projection distortion. To address this, an L2-norm metric in Euclidean space is employed to extract the absolute physical chord length of the actual trajectory $d_{real}\left(t\right)$, formulated as follows:

(12) $$d_{real}\left(t\right)=∥P_{t}-P_{0}{∥}_{2}={\left(x_{t}-x_{0}\right)}^{2}+{\left(y_{t}-y_{0}\right)}^{2}$$

The L2-norm model exhibits rotational invariance and converts the two-dimensional translation between the initial and final poses into an overall displacement magnitude. This formulation reduces the influence of lateral deviation on longitudinal odometry calibration compared with single-axis displacement measurements. Applying the correction factor $K_{linear}$ yields the scale-compensated estimated distance $d_{calib}(t)=K_{linear}\cdot d_{real}(t)$. Given the desired test distance $d_{target}$, the position-tracking error is formulated as $e_{d}\left(t\right)=d_{calib}\left(t\right)-d_{target}$. This error is hot-reloaded into the upper-level nonlinear controller, providing high-fidelity, low-drift feedback to support constant-velocity convergence in the closed loop.

### 2.3. Implementation of the Dual-Layer Calibration System and Its Closed-Loop Coordination Mechanism

Extending the established kinematic error analysis and nonlinear calibration control law, a decoupled software architecture guarantees real-time execution and reliability on the physical platform. Operating within the distributed ROS 2 framework, this architecture isolates the upper-level calibration node from its lower-level execution counterpart. The following discourse details the implementation of the lower-layer drift-suppression mechanism and the online parameter hot-reloading workflow.

#### 2.3.1. Adaptive Covariance Switching Mechanism Based on Zero-Velocity-State Identification

In robot localization frameworks, the odometry covariance matrix represents the confidence interval of the dead-reckoning estimate. Conventional low-level driver nodes output fixed, empirically tuned matrices. During stationary phases, motor driver quantization noise, encoder zero-point jitter, and micro-scale static friction release cause backend estimators to misinterpret these fixed high-uncertainty matrices, inducing global pose drift [28]. To resolve this, an adaptive covariance switching mechanism based on zero-velocity-state detection is introduced. Defining the real-time motion-state norm as $∥V_{t}^{b}{∥}_{1}=|v_{x}(t)|+|v_{y}(t)|+|\omega_{z}(t)|$, the high-frequency monitoring of this metric dictates the dynamic switching of the pose covariance matrix $\sum_{pose}$ and velocity covariance matrix $\sum_{twist}$ published to the /odom topic. The switching criteria are formulated as follows:

(13) $$\sum_{pose}(t)=\left\{\begin{array}{cc} \sum_{stop} & ∥V_{t}^{b}∥=0\\ \sum_{move} & ∥V_{t}^{b}∥>0 \end{array}\right.$$

Under dynamic driving conditions, subject to the Mecanum wheel polygonal effect and microscopic slip, the covariance matrix (1) is configured as follows:

(14) $$\sum_{move}=diag(10^{-3},10^{-3},10^{6},10^{6},10^{6},10^{-3})$$

This matrix assigns bounded variances to the planar observable degrees of freedom (x,y,yaw), setting the unobservable dimensions (z,roll,pitch) to large constants ($10^{6}$) to prevent estimator divergence. The meeting condition $||V_{t}^{b}||=0$ indicates a zero-velocity state, triggering the ZUPT mechanism to contract the covariance matrix to minimal values as follows:

(15) $$\sum_{stop}=diag(10^{-9},10^{-9},10^{6},10^{6},10^{6},10^{-9})$$

The covariance parameters in Equations (14) and (15) were not selected solely through empirical trial and error. Their values were determined by considering the ROS 2 odometry covariance convention, the sensor configuration guidelines of the robot_localization package, measured sensor noise, the planar motion model, parameter sensitivity, and the numerical stability requirements of different estimation back-ends. The proposed state model is limited to planar motion and includes horizontal position, heading, and the associated velocity states. Therefore, the vertical position, roll, and pitch are not included in the estimation model. However, these components are still present in the standard 6-DoF covariance matrices used by ROS 2 messages. Large variances are assigned to them so that they have little influence on the estimated planar pose. In Kalman-filter-based estimators, this setting reduces the weight of the corresponding measurements during the state update. In factor graph optimization, it similarly lowers the information weight of the associated residual terms. The purpose of this configuration is therefore to prevent unobservable or unused components from affecting planar pose estimation, rather than to force these states to fixed values.

The selection of covariance values also requires a balance between measurement confidence and estimation stability. For the stationary covariance matrix in Equation (15), excessively small values may impose an overly strong constraint on wheel odometry measurements, causing the estimator to become less responsive during the transition from stationary to moving states and increasing sensitivity to model mismatch. Conversely, excessively large covariance values weaken the correction effect during stationary intervals and reduce the ability to suppress accumulated odometry drift. Therefore, the selected stationary covariance values were determined to provide sufficient confidence in zero-velocity states while maintaining numerical stability.

During motion, wheel odometry is affected by encoder quantization, periodic roller contact in Mecanum wheels, wheel–ground slip, and mechanical vibration. These effects introduce not only random noise but also non-Gaussian disturbances that vary with the robot’s motion state and the surface condition. Therefore, the dynamic covariance matrix in Equation (14) is defined according to the observed noise level of each measurement component, rather than assuming that wheel odometry is less reliable than the IMU in every degree of freedom.

In the adopted measurement model, wheel odometry provides planar linear and angular velocity information, while the IMU supplies high-frequency angular velocity measurements. For the EKF, UKF, and RCKF, the dynamic covariance is used as the measurement noise covariance during the update step. In the graph-based SLAM optimization framework implemented by slam_toolbox, the corresponding uncertainty is propagated over time to define the noise level of the relative pose factors. This component-wise treatment allows the contribution of wheel odometry and IMU measurements to be adjusted according to their respective uncertainties, thereby reducing the influence of abnormal measurements from a single sensor on the fused pose.

The stationary covariance matrix in Equation (15) is assigned small but non-zero positive values. This increases the weight of near-zero velocity measurements when the robot is stationary, while avoiding the unrealistic assumption that these measurements are completely error-free. If the stationary covariance is too large, the zero-velocity information provides insufficient constraint, and pose drift caused by sensor bias, quantization noise, and random walk cannot be effectively suppressed. By contrast, an excessively small covariance may cause the estimator to become overconfident in the zero-velocity measurements and may reduce the numerical conditioning of the innovation covariance, factor noise model, or linearized system matrix.

To balance constraint strength and numerical stability, the stationary variance was selected through parameter sensitivity analysis. Numerical lower bounds and regularization terms were also introduced where required by the specific estimator. These settings allow the zero-velocity condition to constrain the state estimate without making the optimization or filtering process numerically unstable.

The underlying ROS 2 fusion topology incorporates neither an independent zero-velocity pseudo-measurement node nor auxiliary zero-velocity sensor messages. Instead, the architecture executes a covariance adaptation mechanism conditioned strictly on control command states. During active kinematic command states, the existing wheel odometry input /odom applies the dynamic covariance matrix formulated in Equation (14). Conversely, upon both linear and angular velocity commands satisfying the zero-velocity criteria, the fusion-active velocity covariance within /odom transitions to the stationary covariance matrix delineated in Equation (15).

When a zero-velocity command is issued, the planar linear and angular velocities reported by wheel odometry are expected to remain close to zero. Reducing the covariance of these measurements increases their weight in the state estimation process. In the EKF, UKF, and RCKF, this gives the near-zero velocity measurements a stronger influence during the state update. In the graph-based SLAM optimization framework, the same adjustment reduces the assumed uncertainty of the relative motion factors derived from wheel odometry and therefore increases their information weight.

Unlike a conventional ZUPT method that introduces an additional zero-velocity pseudo-measurement into the measurement update step, the proposed strategy modifies the uncertainty assigned to the existing wheel odometry observations. It therefore imposes a similar zero-velocity constraint without changing the original sensor interface or adding a separate measurement source.

The effectiveness of this mechanism depends on both the covariance switching threshold and the minimum stationary variance. If the stationary covariance is too large, the near-zero velocity observations provide insufficient constraint and static pose drift may remain. If it is too small, the estimator may place excessive confidence in these observations, which can lead to poor numerical conditioning. The selected stationary variance therefore reflects a balance between the strength of the zero-velocity constraint and numerical stability. For this reason, the covariance is kept small but non-zero, and the method does not assume that one parameter setting will remain numerically stable for every possible fusion architecture.

#### 2.3.2. Nonlinear Relay Control Strategy with an Introduced Tolerance Dead Zone

Closed-loop pose error elimination requires incorporating the nonlinear physical characteristics of chassis drive actuators into the control law. The spatial dimension-reduction observation and incremental integration model yield real-time estimates of the longitudinal displacement error $e_{d}(t)$ and accumulated rotational angle error $e_{\theta }(t)$. During robot closed-loop calibration, actuator nonlinearities, including wheel–ground friction and motor dead-zones, dictate the final accuracy. With conventional linear proportional controllers, the control output vanishes as the platform approaches the target, inducing friction-driven steady-state stagnation and residual calibration errors. Furthermore, communication delays and mechanical imperfections trigger high-frequency limit-cycle oscillations near the target equilibrium.

To overcome these physical constraints, a nonlinear relay control strategy featuring a tolerance dead zone replaces the conventional linear paradigm for $e_{d}\;(t)$ and $e_{\theta }(t)$. Defining the state error as $e(t)\in \{e_{d}(t),e_{\theta }(t)\}$ and the control output as $\mu_{cmd}(t)\in \{v_{cmd}(t),\omega_{cmd}(t)\}$, the control law is formulated as follows:

(16) $$\mu_{\mathrm{cmd}}(t)=\left\{\begin{array}{c} U_{set}\cdot \mathrm{sgn}(e(t)),\\ 0, \end{array}\right.\begin{array}{c} |e(t)|>\varepsilon \\ |e(t)|\leq \varepsilon \end{array}$$

In this formulation, $U_{set}$ denotes the constant reference test velocity, corresponding to either the commanded linear velocity $V_{set}\;$ or angular velocity $\omega_{set}$. The sign function sgn(⋅) extracts real-time error polarity. The convergence tolerance deadband ε is defined by the linear position tolerance $\varepsilon_{d}$ and the angular position tolerance $\varepsilon_{\theta }$.

To avoid oscillatory behavior near the target pose, both the relay velocity $U_{set}$ and the deadband ε must be selected appropriately. The relay velocity parameters $U_{set}$ were determined through experimental evaluation of the robot’s motion characteristics. The commanded velocity must be sufficiently large to overcome static friction while remaining low enough to prevent excessive overshoot during stopping. Based on these considerations, the calibration linear velocity was set to $V_{set}\;$ = 0.2 m/s and the angular velocity to $\omega_{set}$ = 0.5 rad/s.

The minimum deadband size is constrained by the discrete-time behavior of the control system. Since the calibration node operates at 20 Hz, the robot should not be able to move completely across the tolerance region within a single control cycle. Therefore, the deadband must exceed the maximum displacement achievable in one sampling interval, together with a small compensation margin $d_{brake}$, resulting in the constraint described by $\varepsilon >U_{set}\cdot T_{s}+d_{brake}$. Using the selected control parameters, the maximum longitudinal displacement per cycle is $\varepsilon_{d}=0.2\;m/s\;\times 0.05\;s=0.01\;m$, while the maximum angular displacement is 0.5 rad/s×0.05 s≈1.43°. Based on these limits, the final deadband values were chosen as $\varepsilon_{d}=0.03\;m$ and $\varepsilon_{\theta }=1.5{}^{\circ}$.

The linear tolerance was slightly increased to 0.03 m to account for residual motion caused by mechanical inertia after a stop command is issued. This allows the robot to settle within the target region without repeatedly crossing the deadband boundary. Similarly, the angular tolerance was increased slightly beyond the theoretical limit to 1.5° in order to accommodate encoder quantization effects and pose estimation noise. This reduces false convergence detections and prevents unnecessary restart oscillations caused by small fluctuations in the measured heading.

The nonlinear control strategy employs a piecewise mapping mechanism to overcome conventional linear control limitations near relay switching. Prior to error convergence ($|e\left(t\right)|>\varepsilon$), the controller bypasses proportional modulation, directly commanding the chassis with a full-amplitude reference velocity dictated by the sign function. This bang–bang output mechanism maintains constant motor torque across both tracking and overshoot-recovery phases. Sustained high torque overcomes wheel–ground static friction, suppressing low-speed creeping and stagnation to yield rapid pose convergence. Upon entering the dead-zone boundary ($|e\left(t\right)|>\varepsilon$), the control law terminates motor power output. Foregoing residual error correction, the system relies on the inherent mechanical damping of the Mecanum-wheeled chassis for a smooth halt. This convergence state triggers a lower-layer distributed service call for online hot reloading of the optimally estimated parameters $K_{linear}$ or $K_{angular}$, finalizing the observation-control-calibration closed loop.

**Figure 4:**
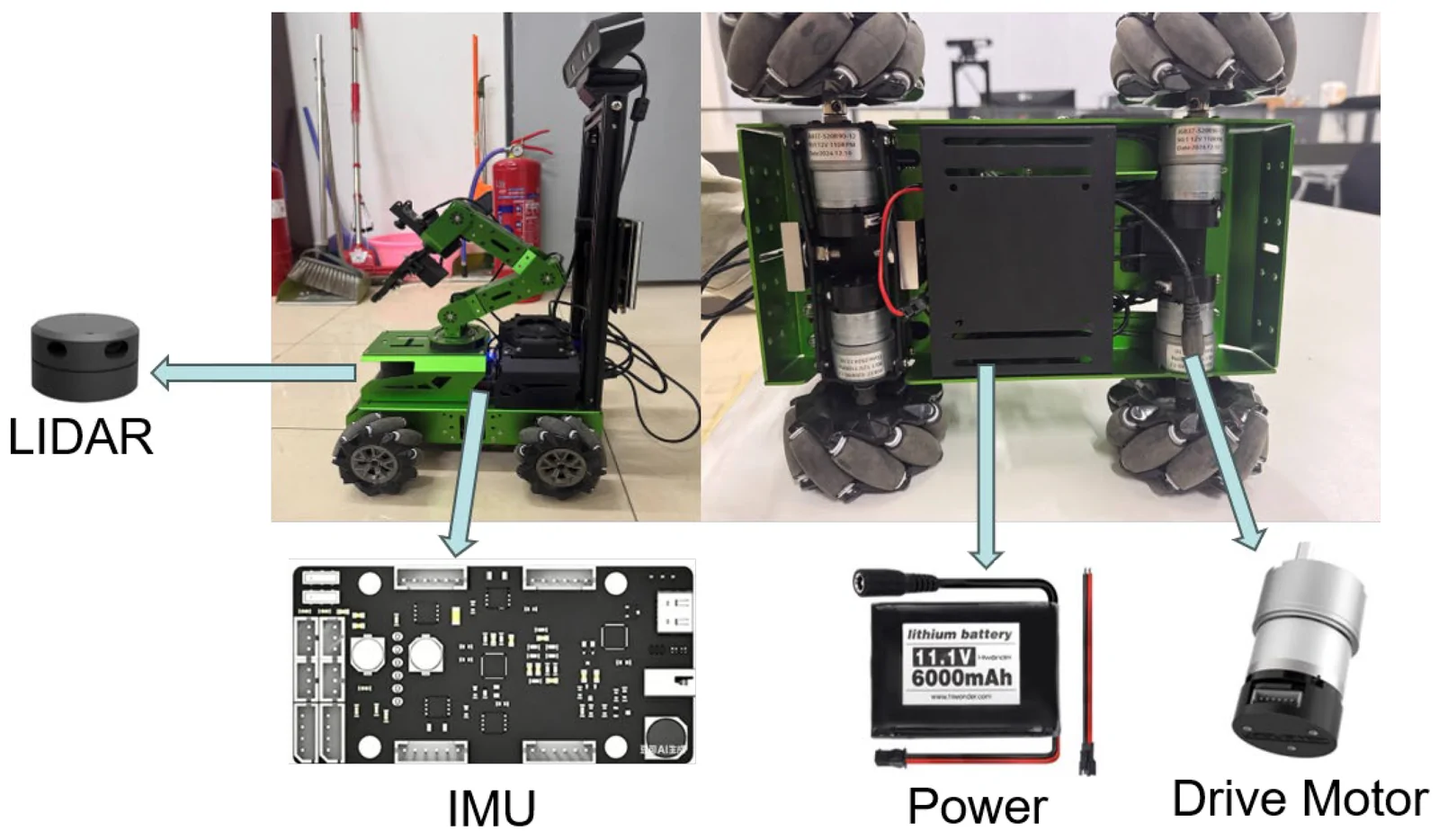
Hardware architecture of the Mecanum-wheeled mobile robot, detailing the core components: LiDAR, IMU, power supply, and drive motors.

Experiments were conducted using a four-wheel omnidirectional Mecanum robot platform (Shenzhen Hiwonder Technology Co., Ltd., Shenzhen, China) to evaluate the proposed odometry calibration framework. The robot has a total mass of 10.5 kg, a wheel radius of 5 cm, a track width of 19.5 cm, and a wheelbase of 21.6 cm. Low-level motor control is implemented on an STM32 microcontroller (Shenzhen Hiwonder Technology Co., Ltd., Shenzhen, China), which incorporates an STM32F407VET6 microcontroller (STMicroelectronics, Geneva, Switzerland) and YX4055 motor-driver chips. The controller drives four DC geared motors, with wheel-velocity feedback provided by Hall encoders integrated into each motor. An MPU6050 six-axis IMU (InvenSense, Inc., San Jose, CA, USA) is used for inertial sensing, with data sampled at 186 Hz. The software system is built on ROS 2 Humble. Within the ROS 2 framework, the odometry estimation and ZUPT-based covariance adaptation modules run at 50 Hz, while the high-level calibration controller operates at 20 Hz. All experiments were conducted on near-horizontal surfaces. Depending on the experimental group, the tested surface was ceramic tile, epoxy resin, or asphalt. Translational ground truth was obtained using a Leica DISTO D5 laser distance meter (Leica Geosystems AG, Heerbrugg, Switzerland), which has a measurement range of 0.05–200 m and a stated accuracy of ±1 mm. Attitude-reference data were obtained from the MPU6050 IMU using its three-axis accelerometer and three-axis gyroscope. The hardware architecture of the experimental platform is shown in Figure 4.

## 3. Results

### 3.1. Multi-Surface Bidirectional In-Place Rotation Calibration Using Normalized Angular Increment Integration

**Figure 5:**
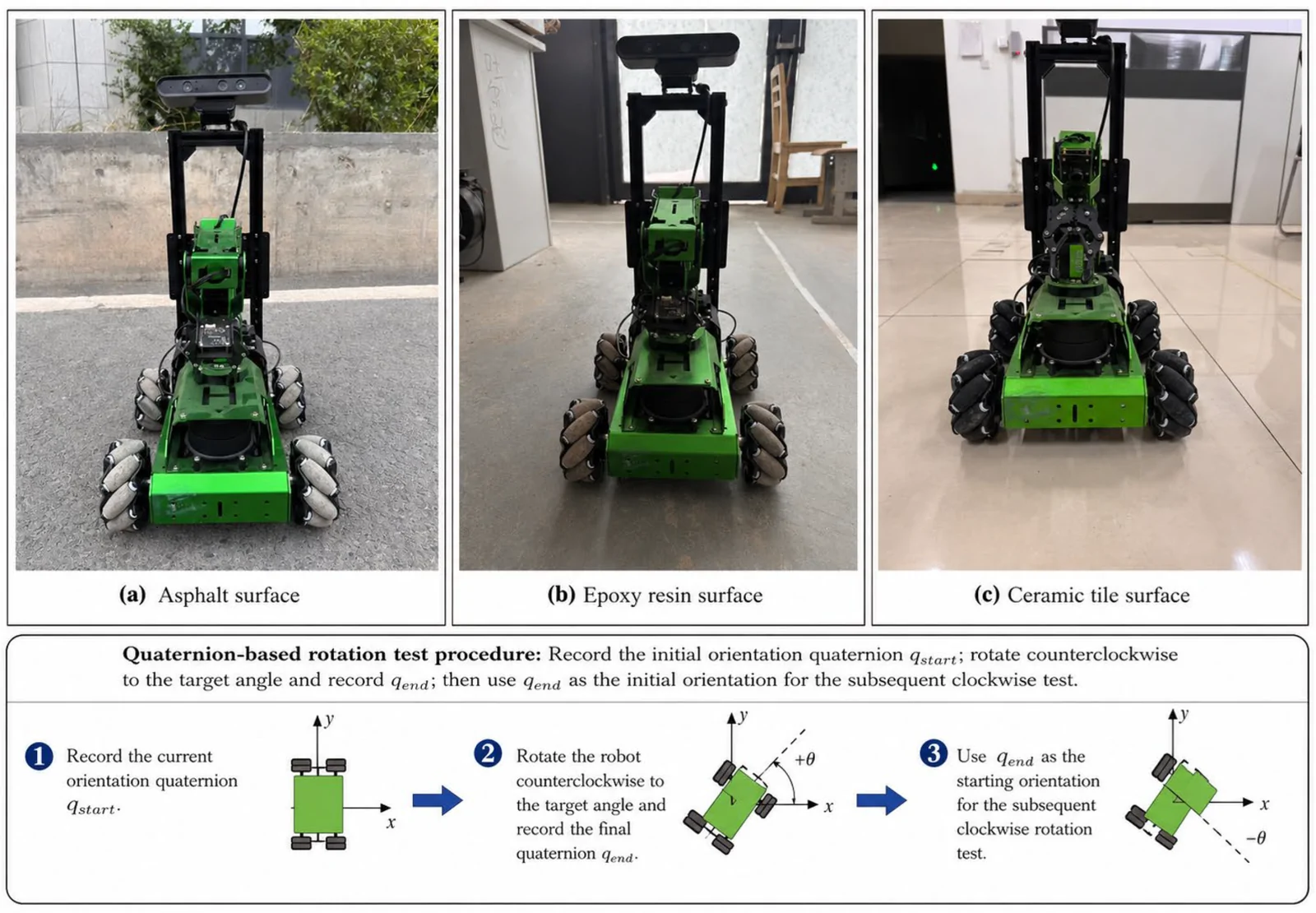
(**a**) Asphalt surface; (**b**) epoxy resin surface; (**c**) ceramic tile surface. The flowchart below illustrates the rotation test procedure. First, the initial attitude quaternion, $q_{start}$ is recorded. The robot is then commanded to rotate counterclockwise to the target angle, at which point the terminal quaternion, $q_{end}$ is recorded. This terminal quaternion is subsequently used as the initial attitude quaternion for the following clockwise rotation test.

Bidirectional in-place rotation tests were conducted at several target angles on three different surfaces: ceramic tile, dust-covered epoxy resin (hereinafter referred to as epoxy resin), and asphalt. These experiments were used to verify that the normalized angular increment method can avoid yaw discontinuities at the ±π boundary and to assess the effectiveness of the closed-loop calibration in reducing rotational scale errors in Mecanum-wheel odometry. The three surfaces produce different wheel–ground interactions and therefore provide a basis for evaluating the stability of the calibration method under varying friction and slip conditions. Figure 5 shows the experimental setups on the three surfaces, together with a schematic of the quaternion-based rotation test procedure.

In each rotation trial, the initial attitude quaternion, denoted as $q_{start}$, is recorded before the robot begins rotating counterclockwise toward the specified target angle. After the robot comes to a complete stop, the terminal attitude quaternion, denoted as $q_{end}$, is recorded. Both quaternions are obtained from the orientation field of the /imu topic.

To maintain continuity between the counterclockwise and clockwise tests and to avoid additional errors caused by repeatedly returning the robot to its initial heading, the terminal quaternion $q_{end}$ from the preceding counterclockwise test is used as the initial quaternion for the subsequent clockwise test. Therefore, the robot is not reset to an absolute zero heading before the clockwise rotation. Instead, the terminal orientation of the previous test is treated as the starting orientation for the reverse rotation. This procedure is consistent with the continuous attitude changes that occur during closed-loop calibration and prevents additional reset motions from affecting the statistical analysis of the rotational errors.

Target rotation angles of 90°, 180°, and 360° were used for both counterclockwise and clockwise rotations. Tests were conducted before and after calibration on each surface. For every combination of surface type, rotation direction, target angle, and calibration state, 20 independent trials were performed. This resulted in 240 rotation trials 3 × 2 × 2 × 20 = 240 for each surface and a total of 720 trials across the three surfaces.

The angular tolerance deadband of the closed-loop rotation controller is defined as $\varepsilon_{\theta }=1.5{}^{\circ}$. To avoid yaw discontinuities at the ±π boundary, the rotation angle is not calculated by directly subtracting the absolute headings at the initial and final poses. Instead, the yaw angle is obtained from the attitude quaternion at each sampling instant, and the normalized angular increments between consecutive samples are accumulated. At the k-th sampling instant, the robot attitude is represented by the quaternion $q_{k}={\left[\begin{array}{cccc} q_{x,k} & q_{y,k} & q_{z,k} & q_{w,k} \end{array}\right]}^{T}$. Its components are arranged as x,y,z,w, following the ROS 2 convention. Under the planar-motion assumption, the yaw angle $\psi_{k}$ is calculated using Equation (17).

(17) $$\Delta \psi_{k}=atan2[sin(\psi_{k}-\psi_{k-1}),cos(\psi_{k}-\psi_{k-1})]$$

For a single rotation trial, the accumulated rotation angle at the N-th sampling instant is calculated as $\Theta N=\sum_{k=1}^{N}\psi_{k}$. To use a consistent statistical definition for both clockwise and counterclockwise rotations, the effective rotation angle is defined as $\theta_{mea}=|\Theta_{N}|\frac{180}{\pi }$. Here, $\theta_{mea}$ denotes the accumulated rotation angle, expressed in degrees, obtained by integrating the incremental changes derived from the attitude quaternions. The absolute angular error of an individual trial is then calculated as $e_{\theta ,i}=|\theta_{mea,i}-\theta_{tar}|$, where $\theta_{tar}$ is the specified target angle and $\theta_{mea,i}$ is the accumulated rotation angle measured in the i-th trial. For each experimental condition, the mean absolute angular error $MAE_{\theta }=\frac{1}{n}\sum_{i=1}^{n}e_{\theta ,i}$ is used to evaluate the overall calibration accuracy. Its mathematical definition is given in Equation (18).

(18) $$s_{\theta }=\sqrt{\frac{1}{n-1}\sum_{i=1}^{n}{\left(e_{\theta ,i}-MAE_{\theta }\right)}^{2}}$$

The mean error reduction rate before and after calibration is calculated using Equation (19).

(19) $$R_{\theta }=\frac{MAE_{\theta ,before}-MAE_{\theta ,after}}{MAE_{\theta ,before}}\times 100\%$$

**Figure 6:**
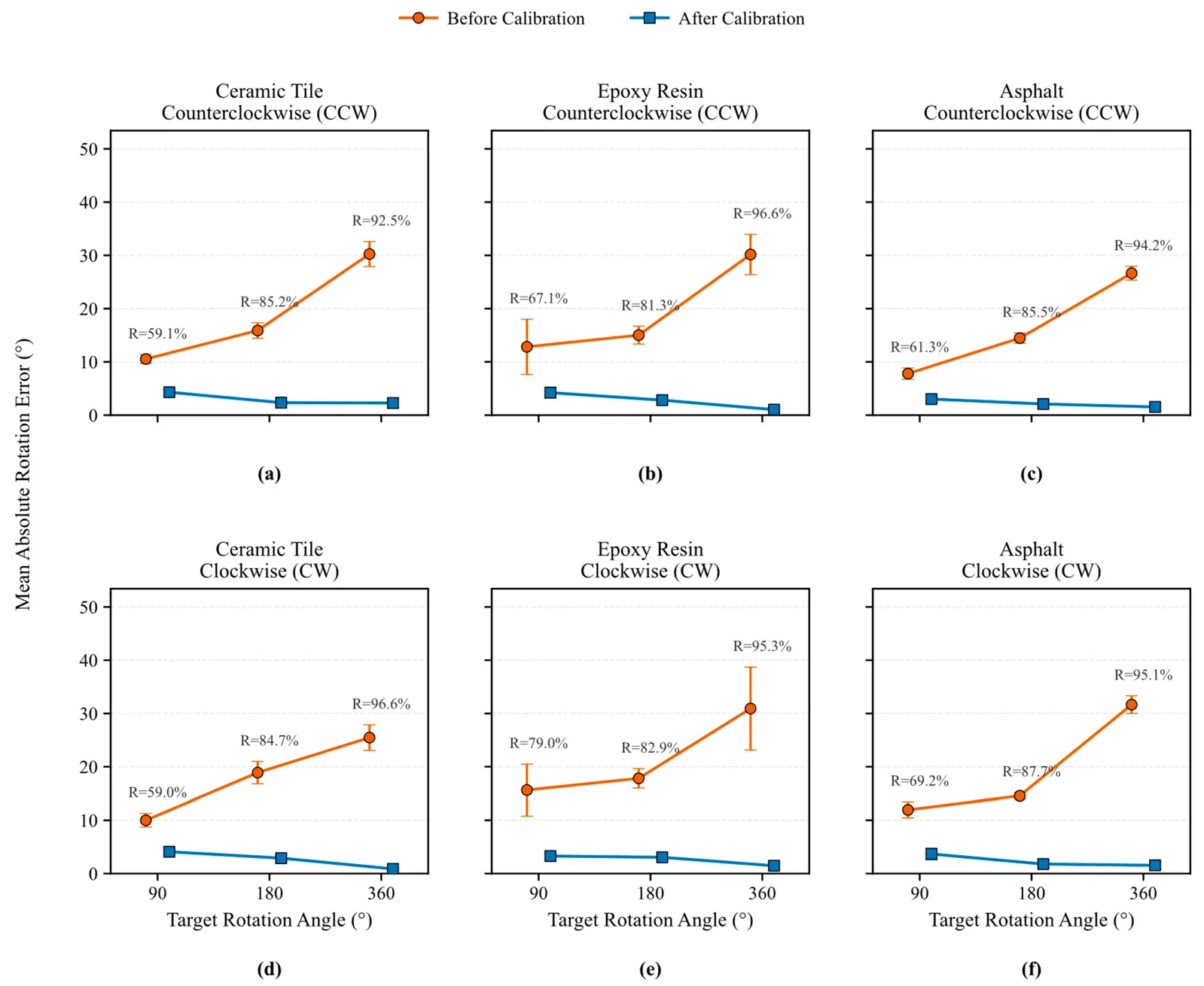
Mean absolute errors obtained from the bidirectional rotation calibration tests on three surfaces: (**a**) counterclockwise rotation on ceramic tile; (**b**) counterclockwise rotation on epoxy resin; (**c**) counterclockwise rotation on asphalt; (**d**) clockwise rotation on ceramic tile; (**e**) clockwise rotation on epoxy resin; and (**f**) clockwise rotation on asphalt. The data points represent the mean absolute rotational errors, and the error bars indicate the (95%) confidence intervals. In addition, R denotes the mean absolute error reduction achieved after calibration.

Figure 6 presents the mean absolute rotational errors and their corresponding 95% confidence intervals for three surface types, two rotation directions, and three target angles. Figure 6a–c show the counterclockwise tests conducted on ceramic tile, epoxy resin, and asphalt, respectively, while Figure 6d–f show the corresponding clockwise tests. The orange circles represent the mean absolute errors before calibration, and the blue squares represent those obtained after calibration. The error bars indicate the 95% confidence intervals of the means, while R denotes the error reduction rate calculated using Equation (19).

**Figure 7:**
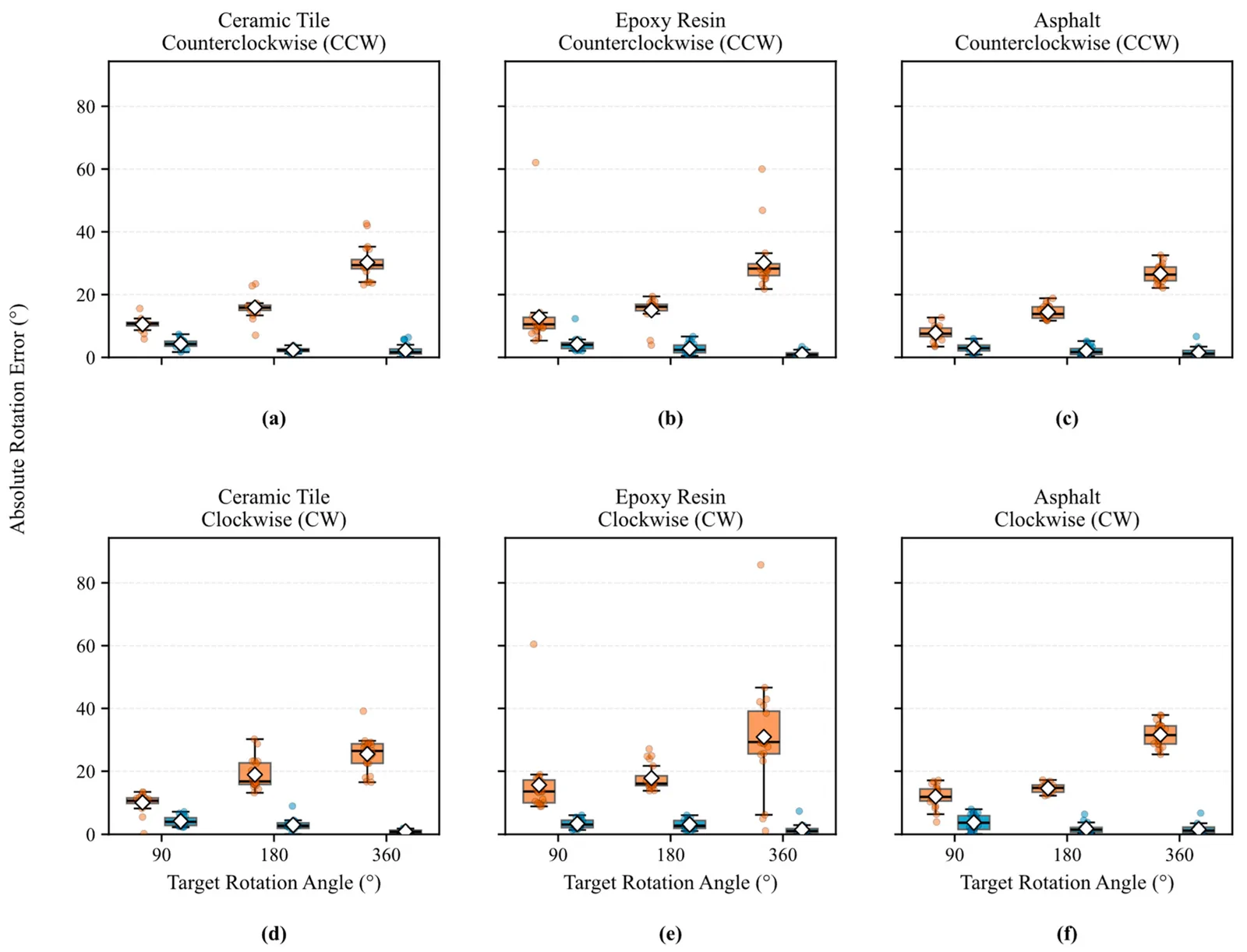
Distributions of the absolute rotation errors obtained from the bidirectional rotation calibration tests on three surfaces. Panels (**a**–**c**) show the counterclockwise tests on ceramic tile, epoxy resin, and asphalt, respectively, while panels (**d**–**f**) show the corresponding clockwise tests. The orange and blue boxes represent the error distributions before and after calibration, respectively. The boxes represent the interquartile ranges, the horizontal lines inside the boxes indicate the medians, and the white diamonds denote the means. The orange and blue scatter points represent the results of the individual trials before and after calibration, respectively.

Figure 7 shows that calibration reduced the mean absolute error for every tested combination of surface type, rotation direction, and target angle. This consistent improvement indicates that the proposed closed-loop calibration method can effectively correct the rotational scale error in Mecanum-wheel odometry. The error reduction rates were lower in the 90° tests than in the 180° and 360° tests. This difference is mainly related to the accumulation of scale error during larger rotations, which gives the compensation factor a more pronounced effect. By contrast, the shorter 90° rotations are more sensitive to startup transients, small wheel–ground slip, and the fixed angular tolerance deadband.

Figure 7 further shows the distributions of the absolute angular errors from all individual trials. In each box plot, the box spans the interquartile range from the first to the third quartile, the horizontal line inside the box indicates the median, and the white diamond represents the mean. The overlaid scatter points show the result of every independent trial. Presenting the central tendency, dispersion, and individual observations together helps reveal abnormal rotations or experimental disturbances that may not be apparent from the mean values alone. To ensure that the experimental results can be traced, the accumulated rotation angles for all trials are reported in Appendix A, Table A1, Table A2, Table A3, Table A4, Table A5 and Table A6. These tables cover the three surfaces, two rotation directions, and three target angles, with each row representing one independent trial.

After correcting the representation of the 360° accumulated angles, the mean rotation angle before calibration was found to exceed the corresponding target angle under every tested combination of surface, direction, and target angle. This consistent positive deviation indicates a systematic tendency toward over-rotation rather than random errors distributed evenly around the target values. For the 360° tests, the mean pre-calibration rotation angles ranged from 385.20° to 391.66°, corresponding to mean absolute errors of 25.47–31.66°. The increase in error at larger target angles suggests that the rotational scale error accumulates with the total rotation angle.

This systematic error is mainly associated with differences between the nominal wheel radius and equivalent track width used in the dead-reckoning model and their effective values during actual rotation. In-place rotation of a Mecanum-wheeled robot is also affected by periodic roller contact, wheel–ground slip, and residual motion during stopping. After closed-loop calibration, the measured rotation angles under all tested conditions moved closer to their target values, indicating that the identified rotational compensation factors effectively reduced the systematic scale error.

Figure 7 also shows that several pre-calibration error distributions had relatively wide ranges and large dispersion. After calibration, the boxes, medians, and means shifted closer to zero, and the overall spread was reduced. These results indicate that the proposed method not only reduces the systematic tendency toward over-rotation but also improves the repeatability of the rotation tests. Overall, the closed-loop calibration framework, together with incremental angular integration, effectively reduces rotational scale errors across the three tested surfaces and brings the measured rotation angles closer to their target values. The detailed statistical results of the bidirectional rotation calibration experiments are summarized in Table 1.

### 3.2. Multi-Surface Longitudinal Straight-Line Calibration Using L2-Norm Displacement Feedback

**Figure 8:**
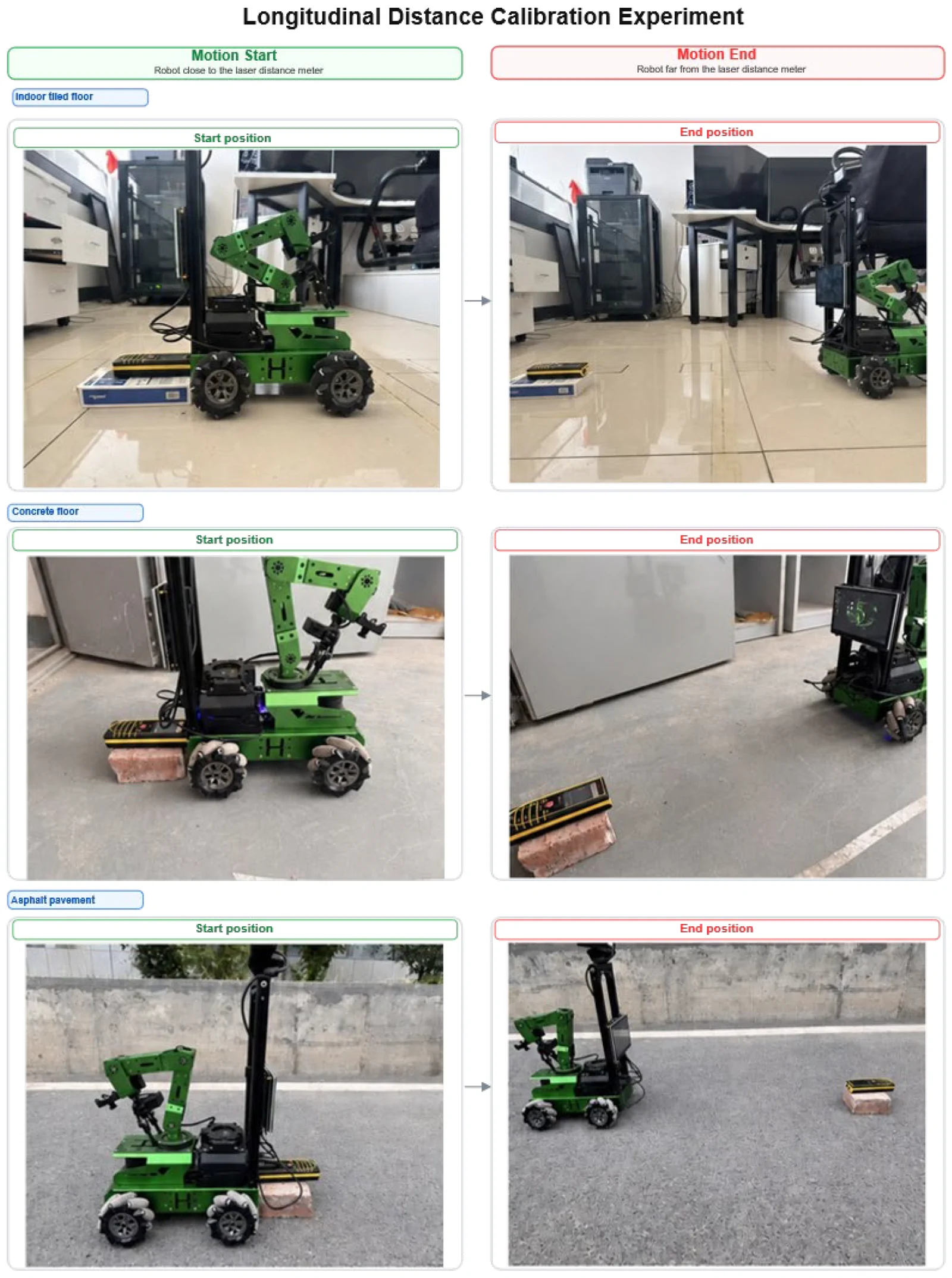
Straight-line driving tests conducted on three different surfaces.

To evaluate the L2-norm-based longitudinal closed-loop odometry calibration method under different wheel–ground interaction conditions, straight-line driving tests were conducted on three surfaces: ceramic tile, epoxy resin, and asphalt. Figure 8 shows the experimental setups and the placement of the laser rangefinder for each surface. The differences in surface roughness, friction, and wheel–ground contact conditions provide a basis for assessing the adaptability of the calibration method under varying terrain conditions.

In each trial, the laser rangefinder was positioned behind the robot and aligned with its longitudinal motion axis. Before the robot began moving, the initial distance reading, denoted as $L_{start}$, was recorded while the robot was stationary. The robot was then commanded to move forward over target distances of 0.5 m, 1.0 m, and 1.5 m. After the robot came to a complete stop, the final distance reading, denoted as $L_{end}$, was recorded. The reference longitudinal displacement for each trial was therefore calculated using Equation (20):

(20) $$d_{ref}=|L_{end}-L_{start}$$

$d_{ref}$ denotes the reference distance measured by the laser rangefinder. Because the instrument was rigidly positioned behind the robot, the difference between the initial and final readings represents the actual longitudinal displacement. To reduce the influence of lateral deviation, which is common in Mecanum-wheeled motion, the estimated odometry distance was not obtained directly from a single coordinate axis. Instead, it was calculated as the $L_{2}$-norm of the displacement between the initial and final planar positions, as defined in Equation (21):

(21) $$d_{odom}=\sqrt{{\left(x_{e}-x_{s}\right)}^{2}+{\left(y_{e}-y_{s}\right)}^{2}}$$

In this formulation, $\left(x_{s},y_{s}\right)$ and $\left(x_{e},y_{e}\right)$ denote the initial and final planar coordinates of the robot trajectory, respectively. The $L_{2}$-norm distance is used as the translational feedback variable in the closed-loop calibration process, reducing the influence of lateral deviation on displacement estimates based on a single coordinate axis. During the experiments, a laser rangefinder positioned behind the robot independently measured the actual longitudinal displacement and provided the reference value for the distance error analysis.

Target distances of 0.5 m, 1.0 m, and 1.5 m were used on each surface, and the tolerance deadband $\varepsilon_{d}$ was set to 0.03 m. Each combination of surface, target distance, and calibration state was repeated 30 times. This produced 180 straight-line driving trials on each surface and 540 trials across the three surfaces. The reference distances were measured using a Leica DISTO D5 laser rangefinder with a stated accuracy of ±1 mm.

All measured distances for the 0.5 m, 1.0 m, and 1.5 m tests on the three surfaces are provided in Appendix B, Table A7, Table A8 and Table A9. These tables report the results obtained before and after calibration and provide the underlying data used to calculate the MAE, SD, and Error Reduction Rate values presented in Table 2.

**Figure 9:**
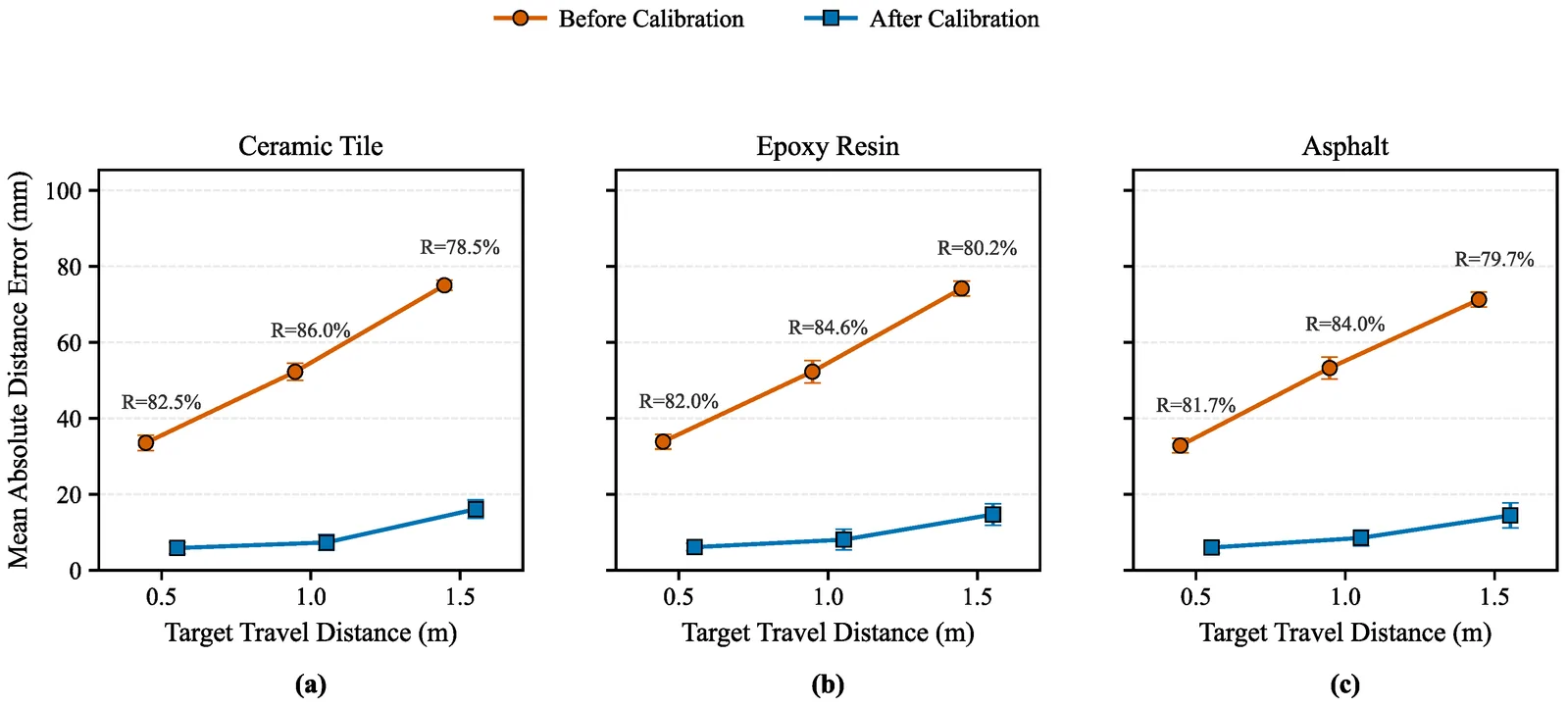
Mean absolute distance errors before and after longitudinal odometry calibration at different target distances on three surfaces: (**a**) ceramic tile; (**b**) epoxy resin; and (**c**) asphalt. The results are presented as mean absolute error (MAE) ± standard deviation (SD), and R denotes the Error Reduction Rate after calibration.

As shown in Figure 9 and Table 2, the mean measured distances before calibration were consistently lower than the corresponding target distances on all three surfaces. This indicates a systematic tendency toward longitudinal under-travel. For target distances of 0.5 m, 1.0 m, and 1.5 m, the mean measured distances ranged from 0.4661 to 0.4672 m, 0.9467 to 0.9493 m, and 1.4250 to 1.4287 m, respectively. The corresponding mean absolute errors were 32.83–33.87 mm, 52.27–53.27 mm, and 71.27–75.03 mm. These results confirm the presence of a longitudinal scale error in the uncalibrated odometry and show that the absolute error increases as the travel distance becomes longer.

For the 0.5 m tests, the mean relative errors before calibration ranged from 6.57–6.77%, which were higher than the ranges of 5.23–5.33% and 4.75–5.00% observed in the 1.0 m and 1.5 m tests, respectively. The larger relative error over shorter distances can be attributed to the greater proportional influence of fixed displacement losses associated with startup friction, actuator deadbands, and deceleration. As the target distance increases, the relative contribution of these transient effects decreases. However, the persistent longitudinal scale error continues to accumulate, leading to a larger absolute error over longer travel distances.

After applying the L2-norm-based closed-loop calibration, the mean measured distances on all three surfaces moved closer to the target values. For the 0.5 m tests, the mean measured distances ranged from 0.4955 to 0.4966 m, corresponding to an under-travel of 3.4–4.5 mm. For the 1.0 m and 1.5 m tests, the mean measured distances ranged from 1.0052 to 1.0061 m and from 1.5145 to 1.5161 m, respectively, indicating slight over-travel at the medium and longer target distances. After calibration, the mean absolute errors decreased to 5.87–6.10 mm, 7.33–8.53 mm, and 14.47–16.10 mm for the 0.5 m, 1.0 m, and 1.5 m tests, respectively. The corresponding mean relative errors remained within 0.73–1.22%.

Across the three surfaces, the Error Reduction Rate ranged from 81.73% to 82.52% for the 0.5 m tests, from 83.98–86.08% for the 1.0 m tests, and from 78.54–80.19% for the 1.5 m tests. The highest reduction, 86.08%, was obtained for the 1.0 m test on ceramic tile, while the lowest reduction, 78.54%, occurred for the 1.5 m test on the same surface. When all nine experimental conditions were considered together, the overall mean absolute error decreased from 53.18 mm before calibration to 9.69 mm after calibration, corresponding to an overall Error Reduction Rate of 82.13%.

**Figure 10:**
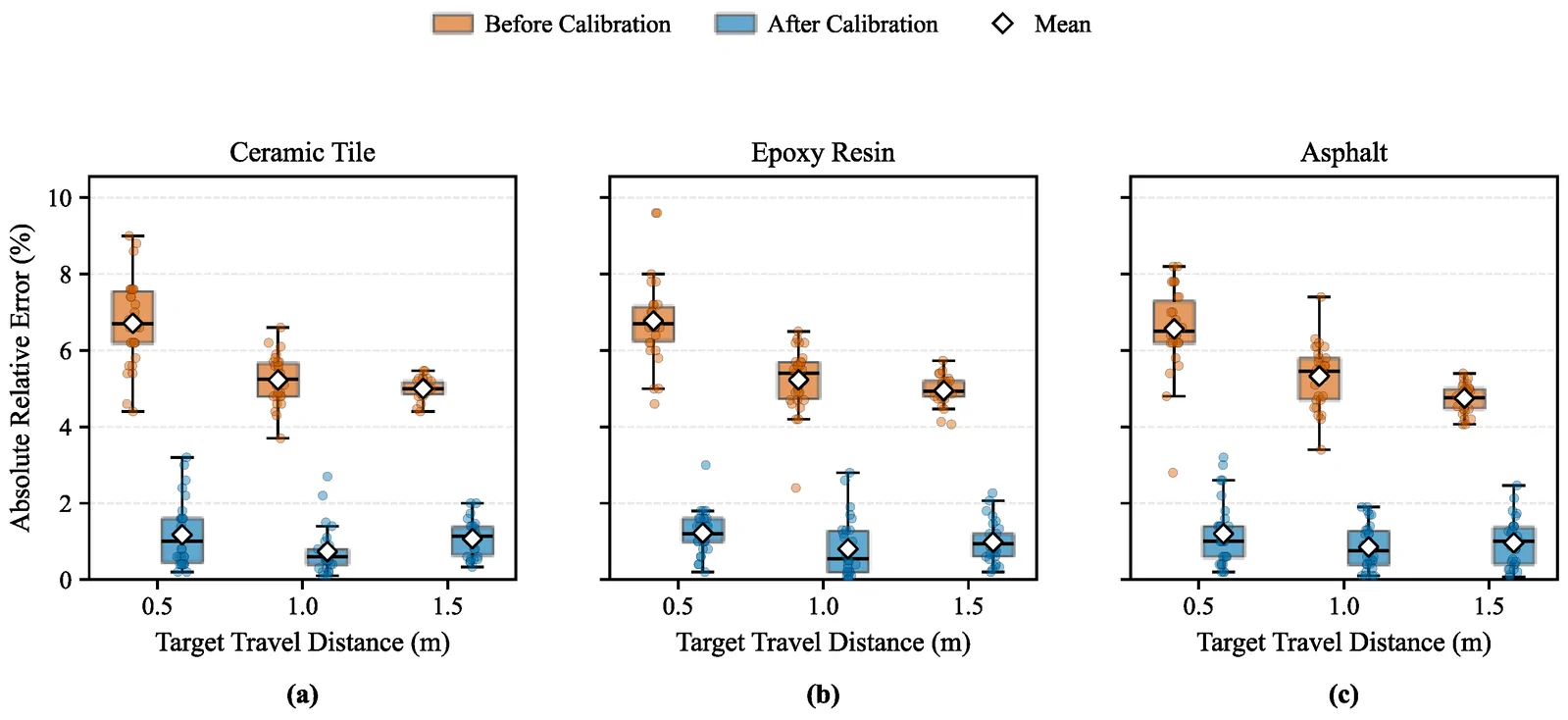
Distributions of the absolute relative errors before and after longitudinal odometry calibration at different target distances on three surfaces: (**a**) ceramic tile; (**b**) epoxy resin; and (**c**) asphalt. The orange and blue boxes represent the error distributions before and after calibration, respectively. The boxes represent the interquartile ranges, the horizontal lines inside the boxes indicate the medians, and the whiskers show the ranges excluding outliers. The orange and blue scatter points represent the results of individual trials before and after calibration, respectively, while the white diamond markers denote the means.

The distributions shown in Figure 10 also indicate that both the medians and interquartile ranges decreased after calibration under all tested conditions. This change suggests that the proposed method reduced the systematic displacement error and improved the repeatability of the experiments. However, the post-calibration error standard deviations for the 1.5 m tests ranged from 6.71 to 9.32 mm, which were higher than the range of 2.76–4.22 mm observed in the 0.5 m tests. This difference indicates that wheel–ground slip, small heading deviations, and variations in the stopping position continue to accumulate as the travel distance increases.

At the same target distance, the three surfaces produced similar mean errors and Error Reduction Rates, indicating that no clear dependence on surface type was observed within the tested conditions. These results show that the L2-norm distance feedback reduces the influence of lateral deviation on displacement estimates derived from a single coordinate axis. The closed-loop calibration also corrects the systematic scale error in longitudinal odometry and provides consistent improvements under the different wheel–ground interaction conditions considered in this study.

### 3.3. Cross-Backend Evaluation of ZUPT-Based Adaptive Covariance Under Intermittent Stop-and-Go Motion

**Figure 11:**
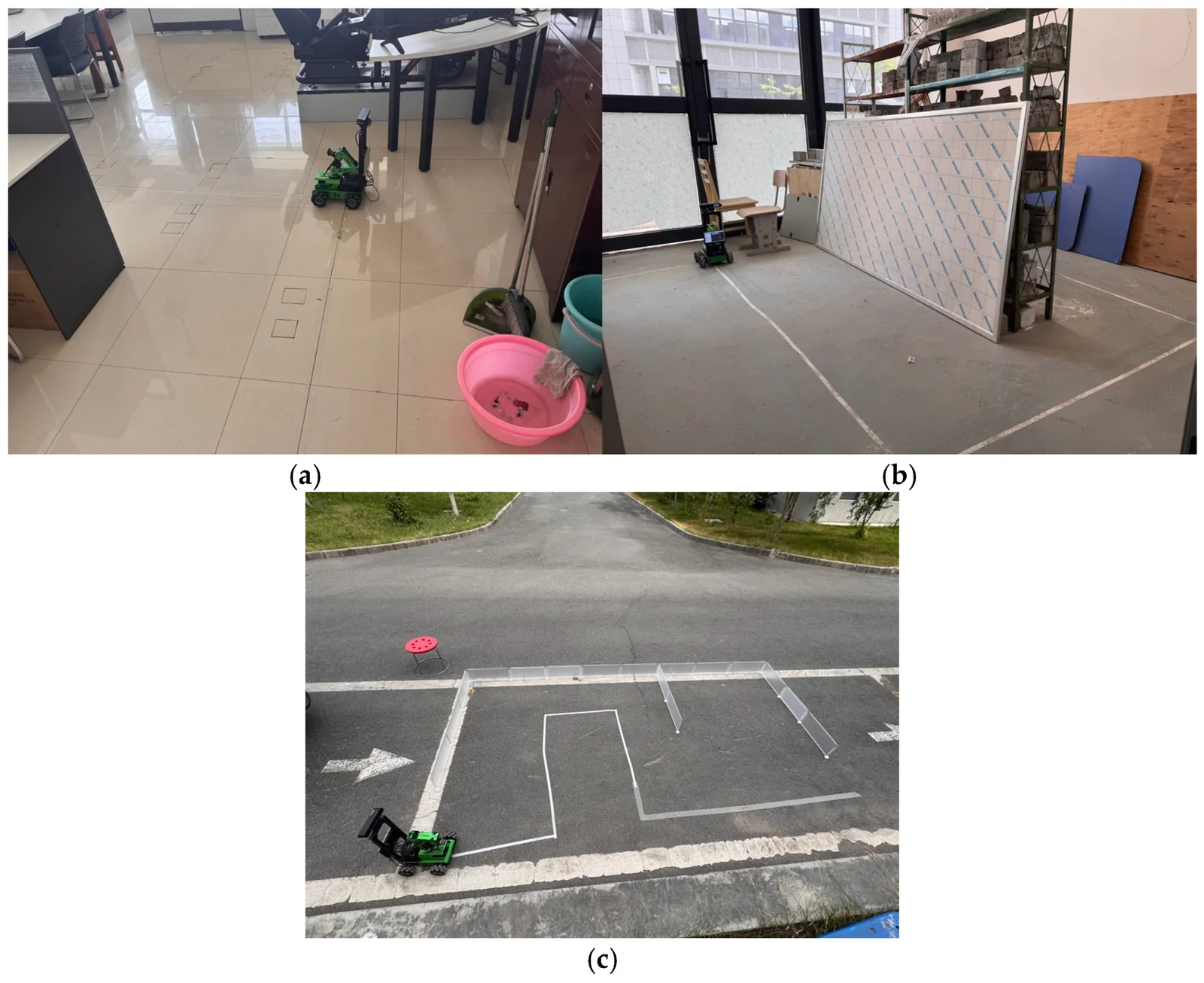
Experimental scenarios for intermittent stop-and-go mapping under different surface conditions: (**a**) ceramic tile; (**b**) epoxy resin; and (**c**) asphalt.

To further evaluate the effect of the proposed Zero-Velocity Update (ZUPT)-based adaptive covariance switching method on pose estimation under different wheel–ground interaction conditions, intermittent stop-and-go mapping experiments were conducted separately on epoxy resin, asphalt, and ceramic tile. Figure 11 shows the experimental environments and surface conditions for the three test scenarios.

Unlike continuous motion at a constant velocity, the designed trajectory consisted of multiple stops, in-place rotations, and subsequent restarts. This protocol was intended to reproduce the intermittent motion patterns commonly encountered in warehouse logistics, routine inspection, and indoor autonomous navigation. At the beginning of each experiment, the robot moved forward from the starting point along a predefined path. Upon reaching each turning point, the robot remained stationary for 20 s, performed an in-place rotation, and then continued along the next path segment. This stop–rotate–move sequence was repeated at each turning point until the robot reached the end of the trajectory.

The 20 s stationary period imposed at each turning point was designed to amplify static pose drift caused by IMU bias, residual wheel-odometry velocity, and sensor noise during standstill. This dwell period also enabled direct evaluation of the ability of the zero-velocity-based covariance adjustment mechanism to suppress erroneous velocity integration. Subsequent restarts and in-place rotations introduced additional dynamic disturbances, including wheel–ground slip, angular-velocity transients, and longitudinal–lateral kinematic coupling. Accordingly, this experimental protocol evaluated the pose estimation accuracy and robustness of the proposed framework under multiple motion states, including complete standstill, in-place rotation, and translational re-acceleration.

To determine whether the improvements achieved through odometry calibration depend on a specific estimation framework, the Extended Kalman Filter (EKF), Unscented Kalman Filter (UKF), Robust Cubature Kalman Filter (RCKF), and a graph-based SLAM optimization framework implemented by slam_toolbox (hereinafter referred to as graph-based SLAM optimization framework) were evaluated using independently collected datasets following the same experimental protocol. These methods were not intended to replace the proposed calibration framework; rather, they were used to examine whether the calibrated odometry consistently improved pose estimation across different estimation paradigms, including recursive filtering, robust nonlinear filtering, and graph-based optimization. Therefore, the experiments focused on the cross-backend applicability of the proposed calibration method rather than establishing a general performance ranking among the selected estimators.

For each surface, six independent intermittent stop-and-go trials were conducted using ROS 2, during which the /pose and /odom topics were recorded simultaneously. The /pose trajectory was generated by slam_toolbox, which derives relative pose constraints from laser scan matching and combines them with odometry and loop-closure constraints to construct a two-dimensional pose graph. The optimized poses are obtained through nonlinear graph optimization. In this study, slam_toolbox was considered as a representative graph-based SLAM optimization framework for evaluating the applicability of the proposed adaptive covariance strategy.

The /pose topic represents the pose estimation result obtained from the graph-based SLAM optimization framework, whereas /odom represents the recursively propagated dead-reckoning pose generated by wheel odometry and the onboard sensor fusion system. Because no external high-precision localization system, such as a motion-capture system or laser tracker, was available, the experiments did not aim to establish an absolute localization ground truth. Instead, the quantitative evaluation focused on the deviation between each pose estimation output and the corresponding odometry trajectory. Therefore, the trajectories generated by EKF, UKF, RCKF, and the graph-based SLAM optimization framework were independently analyzed by calculating their deviations from /odom under both fixed-covariance and ZUPT-based adaptive covariance configurations. This evaluation was designed to verify whether the proposed adaptive covariance strategy can reduce odometry-related drift across different estimation frameworks.

After the six graph-based SLAM trials, 18 additional independent intermittent stop-and-go trials were conducted on each surface for the EKF, UKF, and RCKF evaluations. During every trial, the following ROS 2 topics were recorded simultaneously: /odom, /tf, /tf_static, /cmd_vel, /pose, /scan, and /imu. The /tf and /tf_static topics provide the dynamic and static transformations between the LiDAR, IMU, odometry, and robot base frames. The /cmd_vel topic records the commanded linear and angular velocities and is used to identify motion and commanded zero-velocity periods. The /imu topic provides angular velocity, linear acceleration, and attitude quaternion data, while/scan contains the two-dimensional laser measurements. The /pose topic represents the pose trajectory generated by the graph-based SLAM optimization framework implemented by slam_toolbox, whereas /odom provides the recursively updated dead-reckoning trajectory used as the common comparison baseline in the quantitative evaluation. Recording these topics together preserves the temporal correspondence among motion commands, sensor measurements, coordinate transformations, odometry estimates, and pose-estimation outputs. This allows the same experimental data to be used consistently in the subsequent offline estimation procedures.

For each surface, the 18 datasets were divided into three groups of six independent trials. Each group was assigned to one estimation back-end (EKF, UKF, or RCKF). For each back-end, the corresponding six datasets were processed twice: once using the fixed-covariance configuration and once using the proposed ZUPT-based adaptive covariance configuration. This paired processing ensured that the two covariance strategies were evaluated using the same robot motion, sensor measurements, and pose-estimation outputs. Differences between the resulting /odom-referenced positional deviations could therefore be attributed to the covariance configuration rather than to variations between separate experimental runs.

Because the EKF, UKF, and RCKF were evaluated using three separate groups of experimental data, the results do not constitute a strict comparison of the three filters under identical input conditions. The error values should therefore not be used to establish an absolute performance ranking among the filtering back-ends. Instead, each back-end is evaluated independently by comparing its results under the fixed-covariance configuration with those obtained using the proposed ZUPT-based adaptive covariance configuration. This paired comparison is intended to determine whether the proposed covariance adjustment consistently reduces estimation errors across different state estimation architectures.

Under this experimental design, each surface included six intermittent stop-and-go trials using slam_toolbox and 18 additional trials for the three filtering methods, resulting in 24 independent physical trials per surface. Across the three surfaces, a total of 72 independent intermittent stop-and-go trials were conducted.

After data acquisition, offline pose estimation was performed in a Windows 11 environment using the EKF, UKF, and RCKF. The computing platform was equipped with an Intel Core i7-10875H processor, 16 GB of RAM, and an NVIDIA GeForce RTX 2060 GPU. For each individual back-end, the fixed-covariance and ZUPT-based adaptive covariance configurations were processed using the same raw sensor measurements, time intervals, initial conditions, and corresponding recorded trajectories. Therefore, the comparison was performed within each estimation back-end rather than across different back-ends.

Before deviation calculation, the estimated and reference trajectories were temporally synchronized according to the ROS 2 message timestamps and transformed into a common coordinate frame. Initial-pose rigid alignment was then applied to remove constant translational and rotational offsets caused by differences in coordinate-frame origins. No scale correction was performed, thereby preserving the distance-scale errors inherent in the wheel-odometry measurements. Accordingly, for the k-th synchronized pose, the two-dimensional positional deviation relative to /odom is defined by Equation (22):

(22) $$e_{p(k)}=\sqrt{{\left[\overset{\wedge }{x}(k)-x_{odom}(k)\right]}^{2}+{\left[\overset{\wedge }{y}(k)-y_{odom}(k)\right]}^{2}}$$

wherein, $\overset{\wedge }{x}(k)$ and $\overset{\wedge }{y}(k)$ denote the filter-estimated positional coordinates along the x and y axes, respectively; $x_{odom}(k)$ and $y_{ref}(k)$ denote the corresponding coordinates obtained from the raw odometry trajectory; and $e_{p}(k)$ quantifies the two-dimensional Euclidean distance error between the estimated and corresponding /odom trajectory at the k-th timestamp. The positional deviation relative to /odom is evaluated primarily through the Root Mean Square Error (RMSE) and the Mean Absolute Error (MAE). Specifically, the positional RMSE is governed by Equation (23), whereas the MAE is derived via Equation (24).

(23) $$RMSE=\sqrt{\frac{1}{N}\sum_{k=1}^{N}e_{p}^{2}(k)}$$

(24) $$MAE=\frac{1}{N}\sum_{k=1}^{N}e_{p}(k)$$

where *N* denotes the total number of valid pose pairs retained after temporal synchronization. The root mean square error (RMSE) is more sensitive to large instantaneous positional deviations and therefore reflects the overall trajectory error accumulated throughout the experiment. In contrast, the mean absolute error (MAE) represents the average magnitude of the positional deviations over all synchronized poses, providing a more intuitive measure of the typical pose estimation error. Furthermore, the error reduction rate obtained by replacing the fixed-covariance configuration with the zero-velocity-based adaptive covariance configurations strategy is defined by Equation (25):

(25) $$\eta =\frac{E_{fixed}-E_{ZUPT}}{E_{fixed}}\times 100\%$$

**Figure A1:**
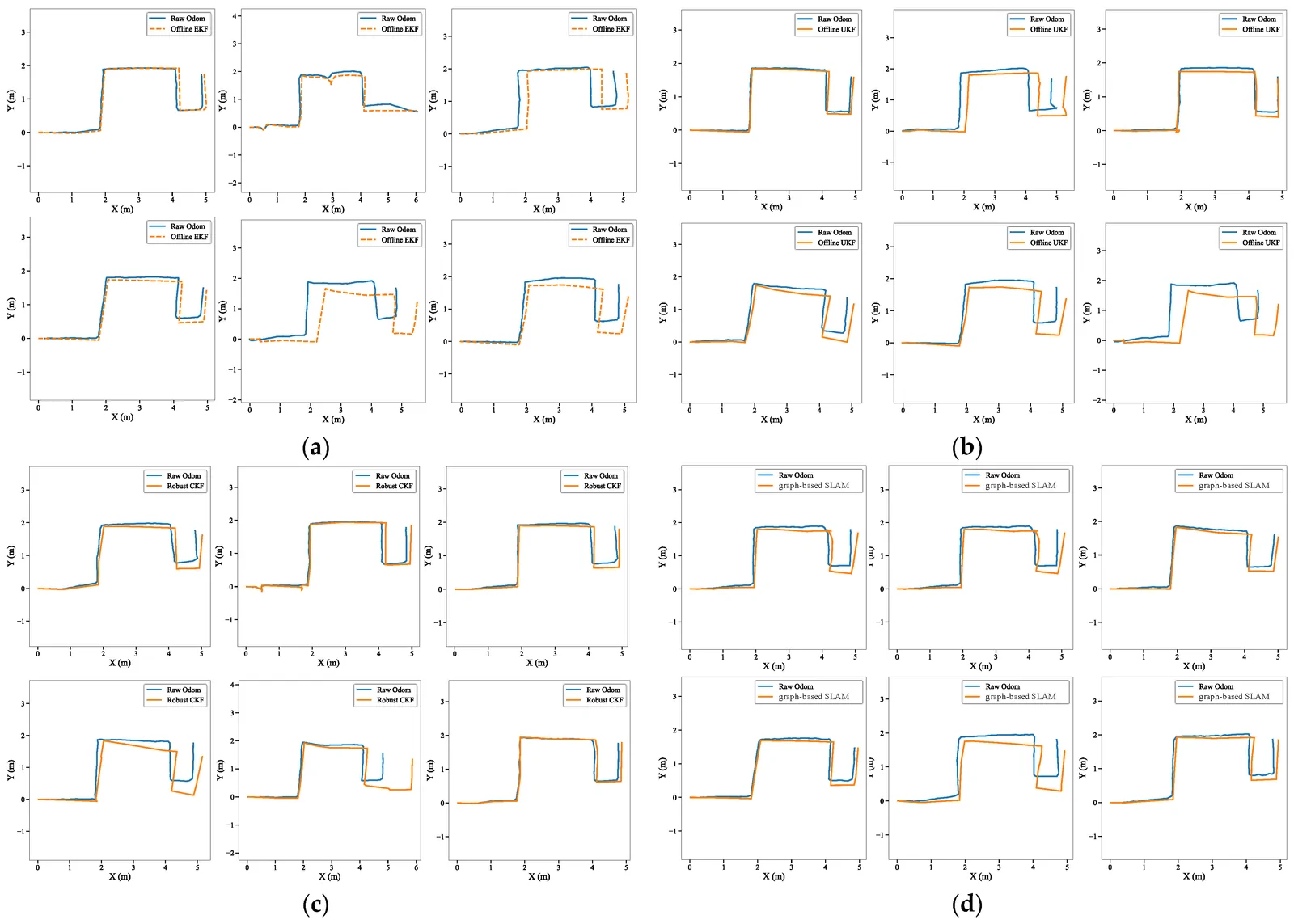
Trajectories obtained with the fixed-covariance configuration on the ceramic tile surface: (**a**) EKF; (**b**) UKF; (**c**) RCKF; and (**d**) the graph-based SLAM optimization framework.

**Figure A2:**
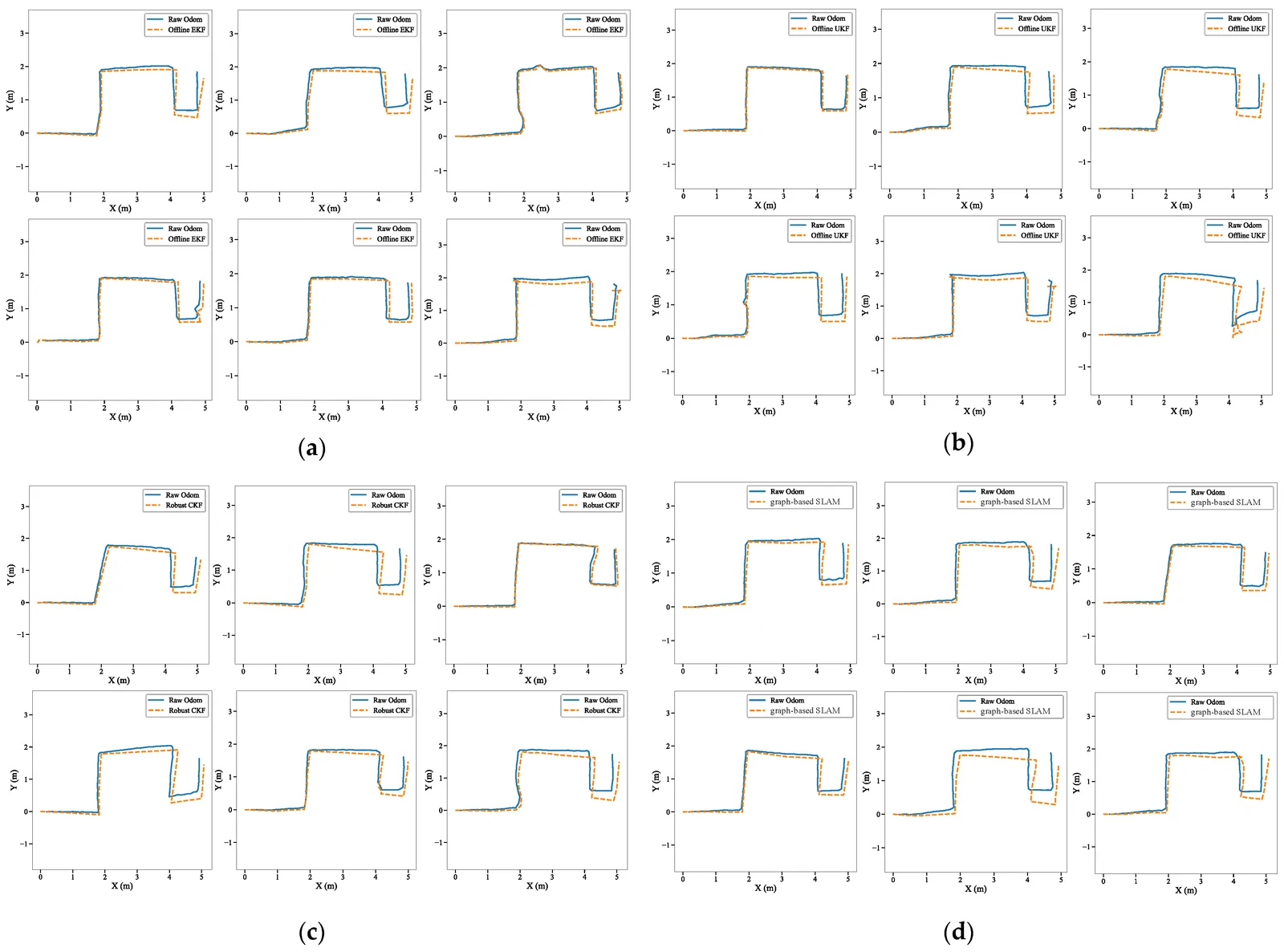
Trajectories obtained with the ZUPT-driven adaptive-covariance configuration on the ceramic tile surface: (**a**) EKF; (**b**) UKF; (**c**) RCKF; and (**d**) the graph-based SLAM optimization framework.

**Figure A3:**
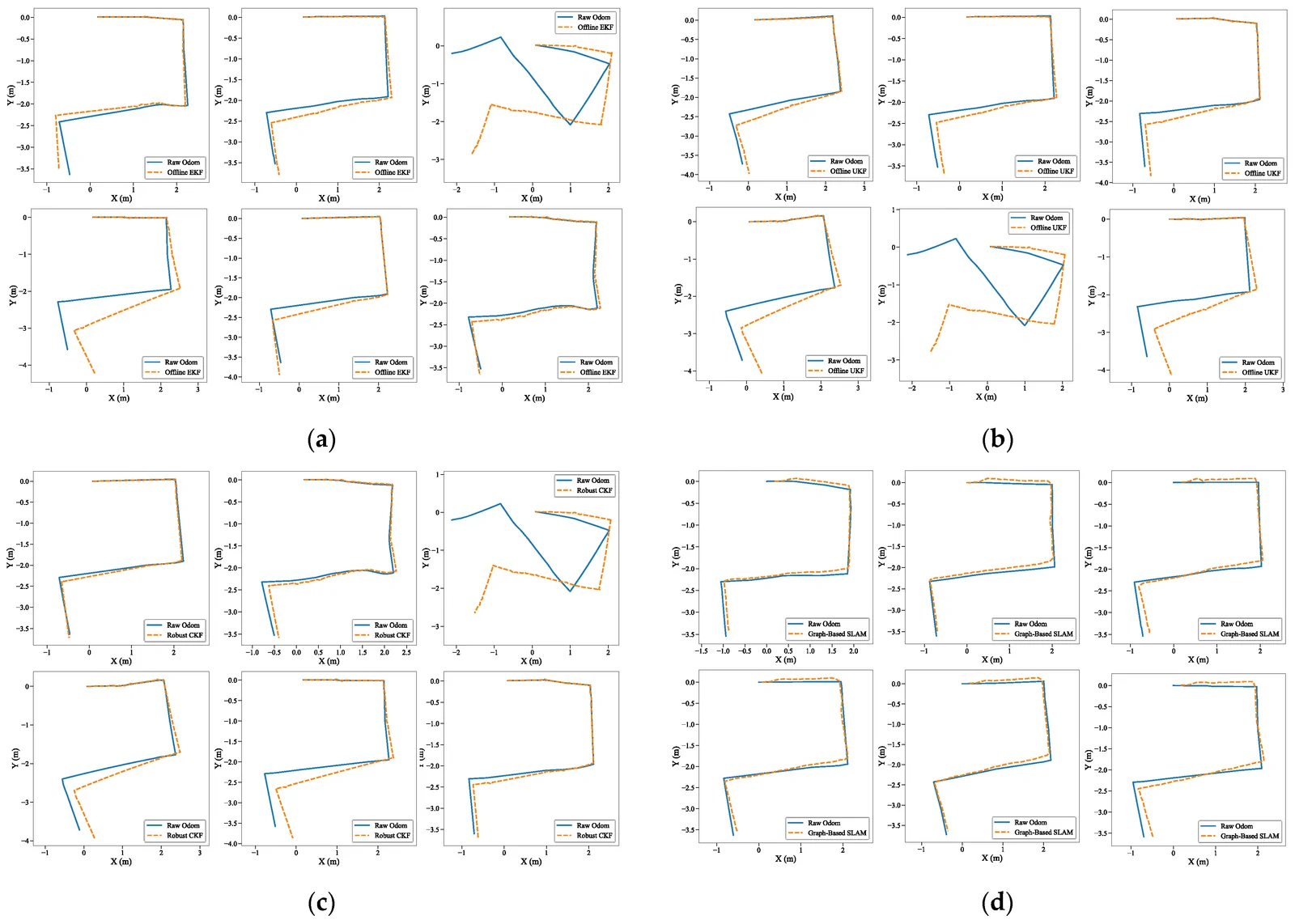
Trajectories obtained with the fixed-covariance configuration on the epoxy resin surface: (**a**) EKF; (**b**) UKF; (**c**) RCKF; and (**d**) the graph-based SLAM optimization framework.

**Figure A4:**
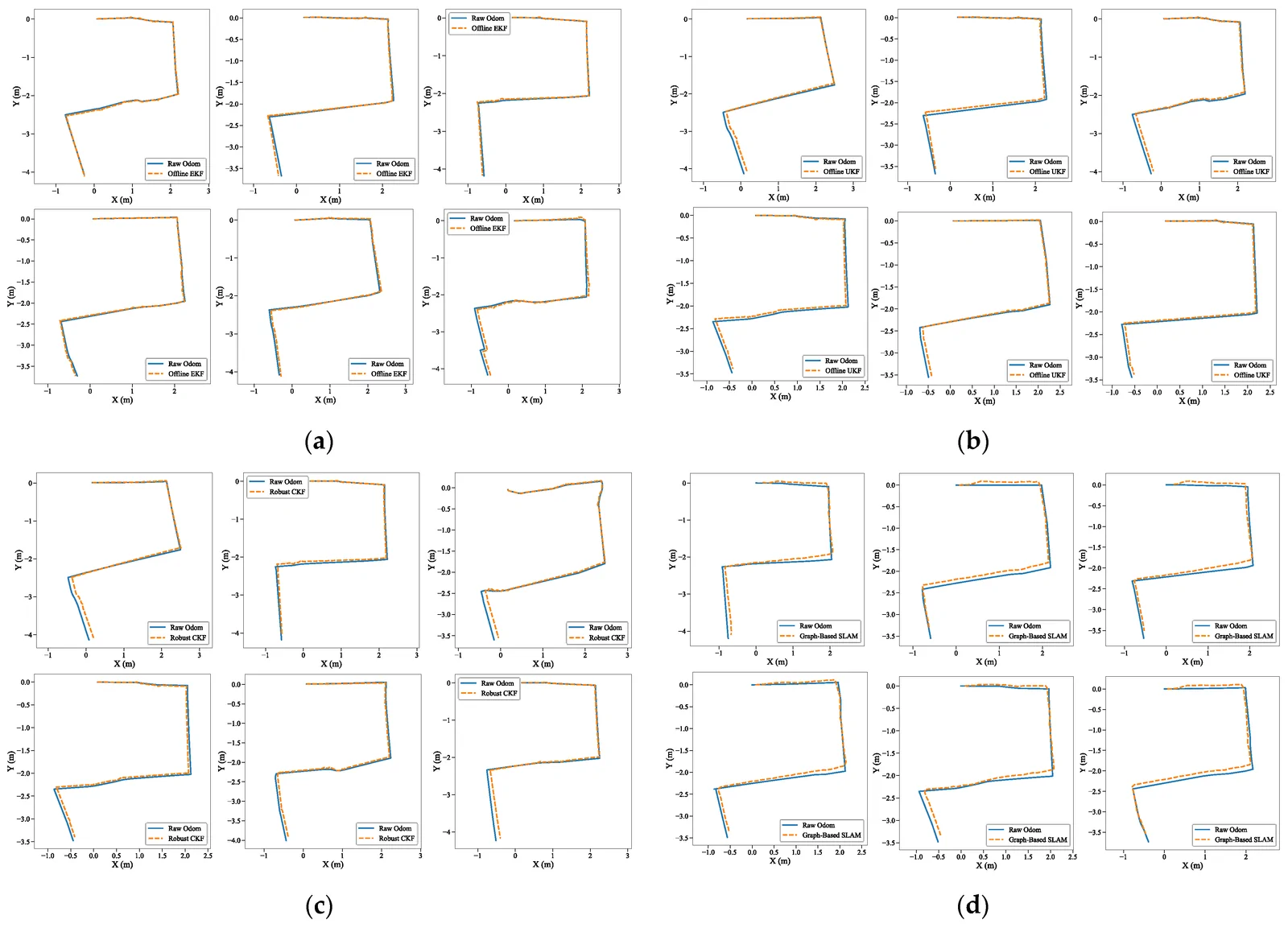
Trajectories obtained with the ZUPT-driven adaptive-covariance configuration on the epoxy resin surface: (**a**) EKF; (**b**) UKF; (**c**) RCKF; and (**d**) the graph-based SLAM optimization framework.

**Figure A5:**
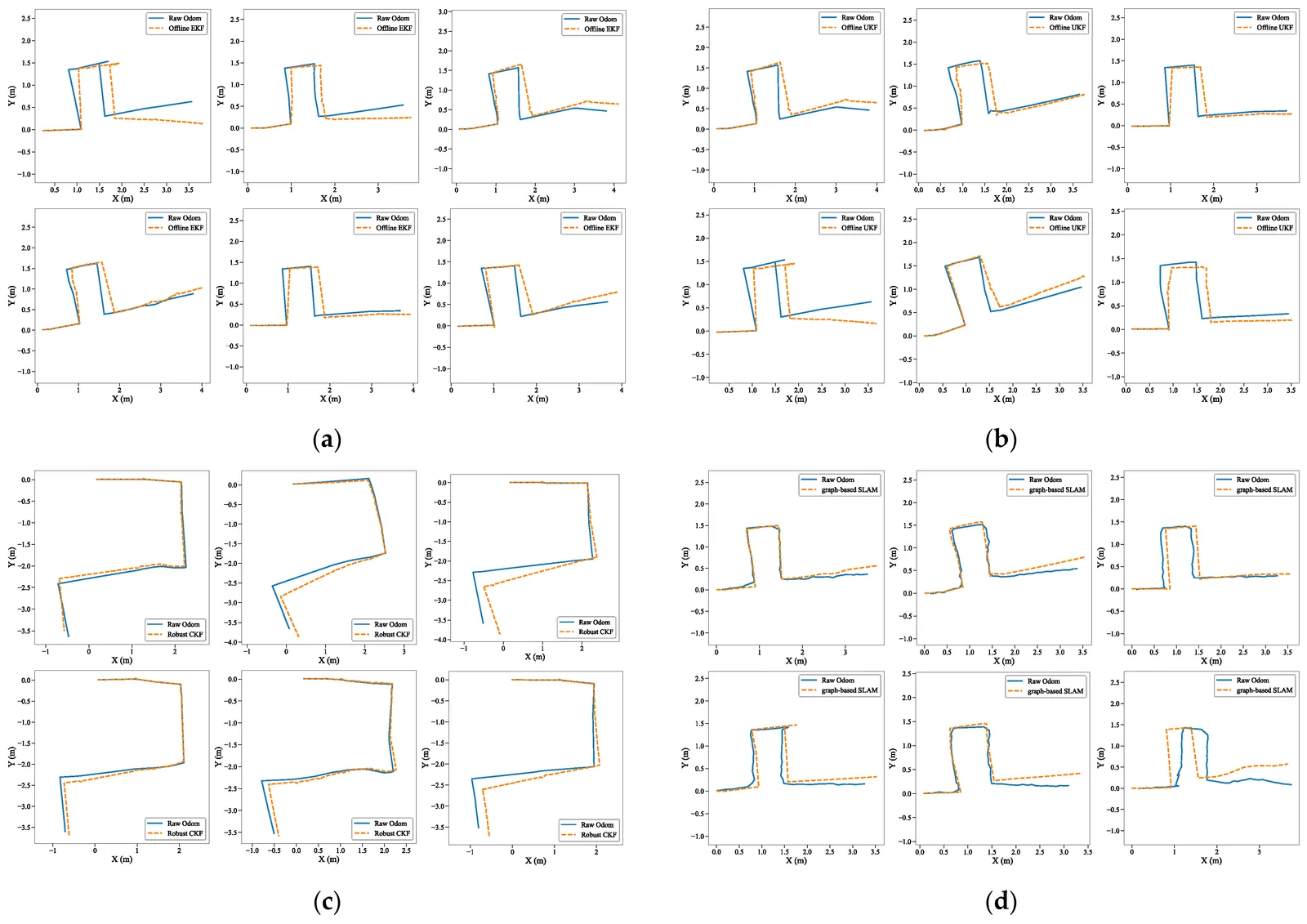
Trajectories obtained with the fixed-covariance configuration on the asphalt surface: (**a**) EKF; (**b**) UKF; (**c**) RCKF; and (**d**) the graph-based SLAM optimization framework.

**Figure A6:**
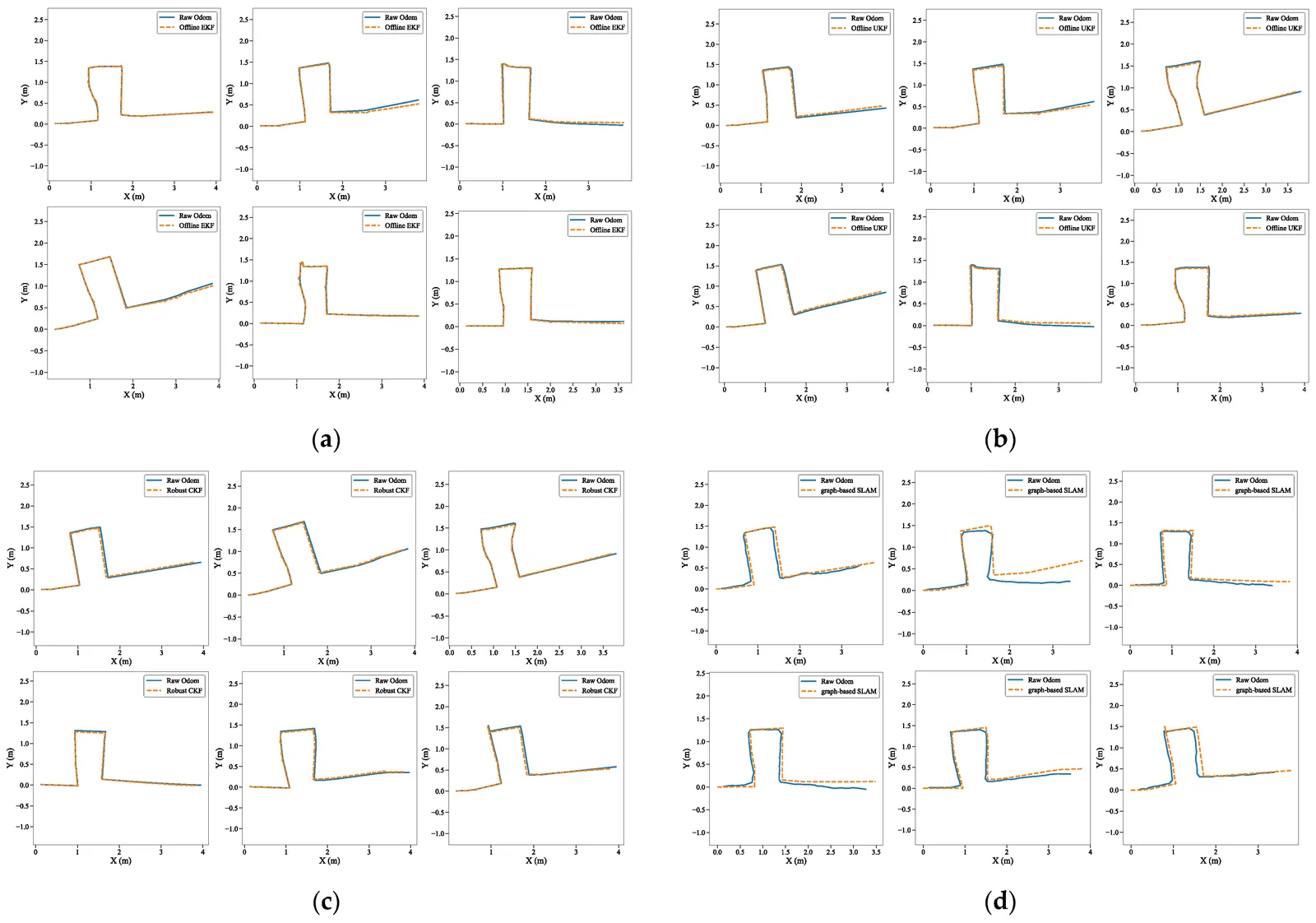
Trajectories obtained with the ZUPT-driven adaptive-covariance configuration on the asphalt surface: (**a**) EKF; (**b**) UKF; (**c**) RCKF; and (**d**) the graph-based SLAM optimization framework.

The group-wise positional error metrics obtained by the four estimation methods under the fixed-covariance and ZUPT-driven adaptive-covariance configurations are reported in Appendix C, Table A10, Table A11, Table A12 and Table A13. The corresponding two-dimensional trajectory comparisons are presented in Appendix C, Figure A1, Figure A2, Figure A3, Figure A4, Figure A5 and Figure A6. Table 3 summarizes the overall error trends observed across the three surface conditions, whereas the appendix provides the complete group-wise numerical results and trajectory profiles as supporting evidence.

As shown in Table 3, the ZUPT-driven adaptive covariance mechanism reduced both positional RMSE and MAE to varying degrees for the EKF, UKF, RCKF, and the graph-based SLAM optimization framework on the ceramic tile, epoxy resin, and asphalt surfaces. These results indicate that the proposed covariance adjustment strategy is not limited to recursive filtering methods but can also be incorporated into graph-based optimization when the odometric covariance is used to define the weighting of motion constraints. By increasing confidence in near-zero odometric velocity measurements during detected stationary periods, the mechanism suppresses pose drift caused by residual wheel-odometry velocities, sensor biases, and measurement noise. The consistent improvements across the four estimation backends therefore demonstrate the cross-backend applicability of the proposed method.

On the epoxy resin surface, the RMSE reduction rates achieved by the EKF, UKF, RCKF, and the graph-based SLAM optimization framework methods were 55.67%, 45.97%, 33.39%, and 19.96%, respectively. Among these methods, the EKF exhibited the largest relative improvement: its RMSE decreased from 0.3603 m to 0.1598 m, while its MAE decreased from 0.3124 m to 0.1529 m, corresponding to an MAE reduction rate of 51.04%.

After incorporating the ZUPT-driven adaptive covariance mechanism, the RMSE values of the UKF and RCKF decreased to 0.1702 m and 0.1582 m, respectively. These results indicate that the proposed mechanism effectively limits the accumulation of positional errors during recursive state estimation, particularly during stationary intervals. Under the fixed-covariance configuration, the graph-based SLAM optimization framework exhibited a relatively low error baseline, with an RMSE of 0.0383 m and an MAE of 0.0344 m. After applying the ZUPT-driven strategy, these values were further reduced to 0.0306 m and 0.0276 m, corresponding to RMSE and MAE reduction rates of 19.96% and 19.74%, respectively.

Although the relative reductions achieved by the graph-based SLAM optimization framework were smaller than those obtained by the three recursive filtering methods, its absolute RMSE and MAE remained the lowest under both covariance configurations. This result suggests that the ZUPT-driven covariance adjustment can further reduce local pose drift during stationary periods even when it is applied to an estimator that already has a comparatively low error baseline. Overall, the results on the epoxy resin surface demonstrate that the proposed mechanism provides consistent benefits across both recursive filtering and the graph-based SLAM optimization framework.

On the asphalt surface, the positional errors of all four estimation methods under the fixed-covariance configuration were consistently higher than those observed on the ceramic tile surface. This difference can be attributed to the greater local roughness of the asphalt, variations in wheel–ground contact conditions, and slip disturbances occurring during in-place rotations and subsequent restarts. Because a Mecanum-wheeled robot interacts with the ground through angled passive rollers, changes in surface flatness and friction can reduce the consistency between the wheel-derived velocity measurements and the robot’s actual motion. These effects increase the uncertainty of the wheel odometry and consequently aggravate the accumulation of dead-reckoning errors.

For RCKF, the RMSE and MAE reduction rates ranged from 19.38% to 67.44% and from 15.20% to 65.95%, respectively. The largest reductions were observed on the ceramic tile surface, whereas the improvements were more limited on asphalt. This variation suggests that the effectiveness of RCKF is influenced by the severity of wheel–ground slip, the prevalence of abnormal measurements, and the degree to which the assumed noise model matches the actual sensor characteristics. In contrast, the graph-based SLAM optimization framework achieved RMSE reduction rates ranging from 19.96% to 53.67%, with the largest improvement occurring on asphalt. This result indicates that graph-based optimization can make effective use of zero-velocity-related odometric weighting to limit accumulated pose errors under more challenging motion and surface conditions.

Across all three surface conditions, the graph-based SLAM optimization framework consistently produced lower positional errors under the fixed-covariance configuration than EKF, UKF, and RCKF. This difference can be explained by the distinct estimation mechanisms of the two categories of methods. Recursive filters update the current state using the preceding estimate and the latest measurements; therefore, positional and heading errors introduced at one time step may propagate through subsequent estimates. In contrast, the graph-based SLAM optimization framework jointly optimizes multiple state nodes and measurement factors over a temporal window, allowing accumulated trajectory deviations to be redistributed and corrected through global or batch optimization.

Despite this inherent advantage, the ZUPT-driven adaptive covariance mechanism further reduced the graph-based SLAM optimization framework RMSE by 19.96%, 53.67%, and 38.13% on the epoxy resin, asphalt, and ceramic tile surfaces, respectively. These results indicate that graph-based optimization alone does not completely eliminate drift associated with prolonged stationary periods and repeated stop-and-go motion. By adjusting the confidence assigned to odometric observations during detected zero-velocity intervals, the proposed mechanism provides more reliable weighting for the corresponding motion constraints and further suppresses local pose drift. The improvements observed across all three surfaces therefore demonstrate that the proposed strategy remains beneficial even when applied to an estimation backend with a comparatively low initial error level.

## 4. Discussion

Despite the effectiveness demonstrated under the tested operating conditions, the proposed method still has several limitations.

Owing to the limited structural strength and vibration-isolation capability of the current robotic platform, preliminary experiments on uneven terrain produced sustained mechanical impacts and chassis vibrations, which damaged the STM32 control board and compromised both equipment safety and the integrity and repeatability of the acquired data. Therefore, localization and calibration performance under markedly uneven surface conditions was not evaluated in the present study. Accordingly, the conclusions derived from the current experiments should not be directly generalized to environments involving severe vertical vibration, repeated wheel–ground impacts, or substantial terrain irregularities.

Because the rotational reference was derived from the onboard IMU rather than an independent external measurement system, the rotation experiments primarily quantify the consistency and relative improvement of the calibrated odometry with respect to the IMU-based attitude reference.

Spatial constraints prevented the construction of an inclined test environment with controllable gradients, sufficiently long trajectories, and repeatable initial conditions. Therefore, the effects of gravitational components, wheel–ground normal-load redistribution, and slope-induced slip on the calibration factors and pose estimation accuracy were not evaluated in this study. The conclusions drawn from the present experiments are consequently limited to near-horizontal surfaces and should not be directly extrapolated to uphill, downhill, or cross-slope motion with the expectation of equivalent error reductions.

In addition, the limited continuous-grasping capability of the onboard manipulator and its insufficient long-term load stability made it difficult to maintain consistent payload placement and center-of-mass distribution across different load conditions. Accordingly, the effects of payload variation on tire deformation, effective rolling radius, wheel–ground friction, and rotational inertia were not systematically investigated. Because the calibration factors used in this study were identified under unloaded conditions, their effectiveness and long-term stability under varying payloads require further experimental validation.

At the algorithmic level, the current covariance update strategy relies on binary switching between motion and stationary states. Although this approach effectively adjusts observation weights during steady motion and standstill, it cannot represent the continuous evolution of state uncertainty during start-up, acceleration, deceleration, and braking. Abrupt switching between two predefined covariance matrices may introduce discontinuities in measurement weighting and consequently reduce pose estimation accuracy during these transitional phases.

Future work will therefore proceed along both hardware and algorithmic directions. At the hardware level, the vibration-isolated mounting of the control board and the damping structure of the chassis will be reinforced. Dedicated test platforms with controllable surface roughness, adjustable gradients, and repeatable payload configurations will also be developed. These facilities will enable compensation parameters to be re-identified under different payload masses, center-of-mass distributions, and impact conditions.

At the algorithmic level, a more robust motion-state recognition method will be developed by jointly using commanded velocity, IMU angular velocity, linear acceleration, encoder feedback, and filtered state estimates. The present binary covariance switching scheme will also be replaced with a continuous adaptive covariance mapping based on motion intensity. Such a formulation is expected to provide smooth covariance adjustment during start-up, steady translation, deceleration, standstill, and motion under external resistance. These improvements will enhance the robustness of the proposed framework under uneven terrain, variable payloads, inclined surfaces, and other highly disturbed operating conditions.

## 5. Conclusions

Empirical results under the tested surface conditions confirm the effectiveness of the proposed framework. In the rotational calibration experiments, which covered three surface conditions, 18 test configurations, and a total of 720 datasets, the absolute angular error was substantially reduced, with error reduction rates ranging from 59.13% to 96.58%. In five of the six 360° conditions, the Error Reduction Rate exceeded 92%, while the asphalt CCW condition achieved a reduction of 88.58%. This result indicates that the rotational error of the uncalibrated system contains a pronounced scale-related bias, which becomes more fully manifested during large-angle rotations. By contrast, small-angle maneuvers are more strongly affected by stochastic disturbances associated with start–stop slip and short-term attitude fluctuations.

In the longitudinal calibration experiments, comprising 540 datasets, the underlying odometry consistently underestimated the actual travel distance. After closed-loop calibration, the mean absolute error across all test conditions decreased from 53.18 mm to 9.69 mm, while the average group-wise error reduction rate reached 82.13%. The similar improvement magnitudes observed across the three surface conditions at the same target travel distances indicate that the identified longitudinal compensation factor maintains consistent calibration performance on the tested near-horizontal surfaces. Nevertheless, the error dispersion under the long-distance condition of 1.5 m remained greater than that observed under the short-distance condition of 0.5 m, suggesting that accumulated wheel slip and stopping-related fluctuations cannot be completely eliminated using a single proportional compensation factor.

Furthermore, the intermittent stop-and-go experiments demonstrate the cross-backend applicability of the ZUPT-driven adaptive covariance mechanism. The EKF, UKF, RCKF, and the graph-based SLAM optimization framework were included not to establish a general performance ranking, but to determine whether the proposed strategy could consistently improve pose estimation across different estimation paradigms. Under all three surface conditions, the adaptive-covariance configuration reduced both RMSE and MAE for all four methods relative to the fixed-covariance configuration, with RMSE reduction rates ranging from 19.38% to 67.44%. The estimation methods generally achieved lower absolute errors on the smooth ceramic tile surface, whereas larger transient deviations were observed on asphalt because of its more complex wheel–ground contact conditions. Nevertheless, the graph-based SLAM optimization framework achieved an RMSE reduction of up to 53.67% on asphalt. This result indicates that the adaptive covariance mechanism can further improve graph-based optimization by assigning more appropriate weights to odometric constraints during detected stationary intervals. Overall, the consistent improvements across the four estimation backends under the tested surface conditions demonstrate that the proposed method is not limited to a specific filtering architecture and can improve the reliability of odometric inputs for different pose estimation methods within the evaluated scenarios.

It should be noted that the experimental validation in this study was conducted on near-horizontal surfaces, including ceramic tile, epoxy resin, and asphalt. The effects of uneven terrain, slope variations, and payload changes were not investigated due to the limitations of the current experimental platform. Therefore, the applicability of the proposed method under these conditions requires further validation in future work.

## Figures and Tables

**Table 1:** Each experimental condition was repeated 20 times. MAE denotes the mean absolute angular error, and SD denotes the standard deviation. CW and CCW indicate clockwise and counterclockwise rotations, respectively.

Surface	Rotation Direction	Target Rotation Angle (°)	Pre-Calibration MAE ± SD (°)	Post-Calibration MAE ± SD (°)	Error Reduction Rate (%)
ceramic tile	CCW	90	10.537 ± 1.898	4.306 ± 1.407	59.13
ceramic tile	CCW	180	15.889 ± 3.334	2.345 ± 0.625	85.24
ceramic tile	CCW	360	30.249 ± 5.357	2.277 ± 1.810	92.47
ceramic tile	CW	90	9.996 ± 2.903	4.045 ± 1.377	59.53
ceramic tile	CW	180	18.895 ± 4.766	2.893 ± 1.732	84.69
ceramic tile	CW	360	25.468 ± 5.475	0.877 ± 0.514	96.56
epoxy resin	CCW	90	12.827 ± 11.809	4.221 ± 2.232	67.09
epoxy resin	CCW	180	15.017 ± 3.800	2.813 ± 1.690	81.27
epoxy resin	CCW	360	30.164 ± 8.622	1.033 ± 0.915	96.58
epoxy resin	CW	90	15.631 ± 11.097	3.834 ± 1.374	75.47
epoxy resin	CW	180	17.817 ± 4.139	3.055 ± 1.413	82.85
epoxy resin	CW	360	26.980 ± 13.116	1.464 ± 1.588	94.57
asphalt	CCW	90	7.795 ± 2.428	3.018 ± 1.337	61.29
asphalt	CCW	180	14.457 ± 2.193	2.094 ± 1.456	85.52
asphalt	CCW	360	26.647 ± 2.956	3.044 ± 7.962	88.58
asphalt	CW	90	11.936 ± 3.411	3.673 ± 2.469	69.23
asphalt	CW	180	14.558 ± 1.422	1.786 ± 1.485	87.73
asphalt	CW	360	31.660 ± 3.790	2.387 ± 1.866	92.46

**Table 2:** Error statistics before and after longitudinal odometry calibration at different target distances on three surfaces.

Surface	Target Distance (m)	Mean Distance Before Calibration (m)	Mean Distance After Calibration (m)	Pre-Calibration MAE ± SD (mm)	Post-Calibration MAE ± SD (mm)	Error Reduction Rate (%)
ceramic tile	0.5	0.4664	0.4955	33.57 ± 5.59	5.87 ± 4.22	82.52
ceramic tile	1	0.9477	1.0052	52.27 ± 6.19	7.33 ± 5.83	85.97
ceramic tile	1.5	1.425	1.5161	75.03 ± 3.79	16.10 ± 6.71	78.54
epoxy resin	0.5	0.4661	0.4966	33.87 ± 5.44	6.10 ± 2.76	81.99
epoxy resin	1	0.9493	1.0061	52.30 ± 8.20	8.07 ± 7.56	84.58
epoxy resin	1.5	1.4258	1.5145	74.20 ± 5.44	14.70 ± 7.81	80.19
asphalt	0.5	0.4672	0.4957	32.83 ± 5.43	6.00 ± 4.16	81.73
asphalt	1	0.9467	1.0059	53.27 ± 8.07	8.53 ± 5.79	83.98
asphalt	1.5	1.4287	1.5145	71.27 ± 5.41	14.47 ± 9.32	79.7

**Table 3:** Positional deviation statistics relative to /odom for different pose estimation methods under three surface conditions.

Surface	Method	Fixed-Covariance RMSE (m)	ZUPT-Based Adaptive-Covariance RMSE (m)	RMSE Reduction Rate (%)	Fixed-Covariance MAE (m)	ZUPT-Based Adaptive-Covariance MAE (m)	MAE Reduction Rate (%)
epoxy resin	EKF	0.3603	0.1598	55.67	0.3124	0.1529	51.04
epoxy resin	UKF	0.3150	0.1702	45.97	0.2793	0.1661	40.54
epoxy resin	RCKF	0.2375	0.1582	33.39	0.2305	0.1539	33.22
epoxy resin	Graph-based SLAM optimization framework	0.0383	0.0306	19.96	0.0344	0.0276	19.74
asphalt	EKF	0.4088	0.2373	41.96	0.3649	0.2257	38.15
asphalt	UKF	0.3947	0.2225	43.62	0.3623	0.2171	40.09
asphalt	RCKF	0.2719	0.2192	19.38	0.2460	0.2086	15.20
asphalt	Graph-based SLAM optimization framework	0.1271	0.0589	53.67	0.1066	0.0509	52.29
ceramic tile	EKF	0.0809	0.0348	57.01	0.0698	0.0313	55.24
ceramic tile	UKF	0.0568	0.0317	44.11	0.0500	0.0284	43.27
ceramic tile	RCKF	0.1042	0.0339	67.44	0.0903	0.0308	65.95
ceramic tile	Graph-based SLAM optimization framework	0.0523	0.0324	38.13	0.0482	0.028691	40.72

## Data Availability

The data presented in this study are available on request from the corresponding author. The data are not publicly available due to ongoing project restrictions.

## Abbreviations

The following abbreviations are used in this manuscript:

ZUPT	Zero-Velocity Update
RMSE	Root Mean Square Error
ROS 2	Robot Operating System 2
IMU	Inertial Measurement Unit
EKF	Extended Kalman Filter
ATE	Absolute Trajectory Error
RCKF	Robust Cubature Kalman Filter

## Appendix A. Raw Rotational Angle Calibration Data

**Table A1:** Raw rotational angle measurements before and after calibration for counterclockwise rotations on the epoxy resin surface.

Trial No.	90°		180°		360°	
	Before Calibration (°)	After Calibration (°)	Before Calibration (°)	After Calibration (°)	Before Calibration (°)	After Calibration (°)
1	98.5911	102.3250	194.9846	181.5618	385.0600	359.9081
2	100.8025	93.5252	194.4697	182.0471	419.9924	358.9875
3	99.3872	94.4067	196.2502	181.1749	406.8367	358.8710
4	100.7215	92.5890	196.2318	185.1597	388.2155	359.5828
5	96.3600	93.1068	194.9817	186.6497	388.0895	359.9276
6	100.7678	92.1166	196.1655	180.9695	386.0127	358.8374
7	102.7834	94.5096	196.8842	181.1288	389.8160	359.9974
8	100.3203	94.8690	195.6225	184.2585	388.7422	358.6247
9	99.6570	92.8943	197.9554	180.4810	387.5039	357.7306
10	99.9550	92.1896	185.3826	183.3432	393.2039	359.4814
11	102.9901	94.2392	199.4202	176.8642	385.0531	359.8295
12	152.0472	95.6951	194.7658	177.4407	383.2597	359.1956
13	95.3386	95.6836	196.0608	177.7331	389.8004	359.1042
14	104.2590	94.5374	193.8990	181.3349	381.7737	356.5641
15	102.7365	92.6663	194.9181	176.2423	388.3570	357.5455
16	99.5104	93.2185	197.9183	182.3849	387.8185	359.1807
17	101.3849	94.6291	196.2314	181.7093	388.4193	359.7907
18	98.4193	92.1093	197.4201	184.2928	386.1049	358.4907
19	102.8928	93.8185	196.8846	177.5849	390.5185	358.0907
20	97.6185	95.2928	183.9019	185.6193	388.6928	359.6072

**Table A2:** Raw rotational angle measurements before and after calibration for clockwise rotations on the epoxy resin surface.

Trial No.	90°		180°		360°	
	Before Calibration (°)	After Calibration (°)	Before Calibration (°)	After Calibration (°)	Before Calibration (°)	After Calibration (°)
1	99.9848	92.7529	195.7225	182.2119	385.3008	359.1353
2	98.7973	95.2046	195.5529	185.9697	387.7996	352.7326
3	107.6412	93.1106	197.4757	184.5381	406.6331	358.8075
4	108.9269	86.5182	196.2705	185.1233	389.9990	359.6347
5	107.7233	93.0476	195.9696	181.5140	361.1183	359.9326
6	107.0384	94.2309	196.9112	184.4463	355.1525	359.3722
7	98.8603	95.7840	203.9244	182.5642	383.2592	359.8218
8	99.5118	92.7263	195.4515	181.3889	353.9056	359.0704
9	100.3421	93.2173	195.3600	182.4708	388.7413	359.4937
10	99.6416	92.7284	196.3745	184.2607	353.0867	357.8523
11	103.9335	94.7794	163.8964	176.8146	400.9249	358.5194
12	150.4528	96.9709	194.7242	178.0846	389.4986	358.2814
13	103.5779	91.8835	195.8214	178.9542	389.0833	359.8532
14	103.5218	95.1768	193.8488	177.5169	388.9955	357.3302
15	103.5392	94.0562	204.7416	175.6746	402.9402	358.8846
16	103.2985	94.1093	194.6273	183.5928	391.4093	359.4072
17	99.8193	92.8184	204.8976	181.9185	402.1049	358.5151
18	106.5928	95.3185	201.6951	178.6849	385.6184	357.8807
19	101.1185	91.5928	207.1016	184.1193	389.8093	359.0072
20	108.3049	93.6849	193.7644	182.7093	398.5185	357.1815

**Table A3:** Raw rotational angle measurements before and after calibration for counterclockwise rotations on the asphalt surface.

Trial No.	90°		180°		360°	
	Before Calibration (°)	After Calibration (°)	Before Calibration (°)	After Calibration (°)	Before Calibration (°)	After Calibration (°)
1	99.3105	93.5634	196.4155	180.3519	383.5203	359.4268
2	97.5721	94.3055	192.6553	178.2480	385.9302	358.2673
3	100.0197	92.1558	197.1810	176.6502	387.4327	359.6263
4	101.8529	92.9690	191.6850	181.6723	388.7432	359.1407
5	97.2458	93.1695	195.9688	178.7157	382.7355	359.7243
6	97.9031	91.4703	198.8273	177.3053	385.4628	358.3305
7	102.6983	92.9427	192.4041	179.5758	385.1431	357.8211
8	99.9398	91.9995	194.0115	178.7116	384.4763	357.7038
9	97.5507	92.2656	195.3436	184.7549	391.4053	356.5955
10	99.2334	91.1800	193.5329	183.0229	386.7993	358.8188
11	96.8744	92.7758	191.9688	181.4780	382.9442	359.9476
12	97.1156	92.9684	195.2108	184.4386	388.9383	358.7879
13	98.7012	94.3610	192.1175	180.9117	392.5298	323.3485
14	93.4344	94.2301	196.0919	179.2895	389.1043	357.7571
15	93.7184	95.0876	192.5816	185.1916	382.1253	357.5702
16	96.7905	90.8728	197.9959	182.4402	385.0600	358.7466
17	95.5335	93.7637	193.5222	177.9939	390.0095	359.0040
18	99.3090	92.9262	193.6369	180.5047	384.3018	359.8044
19	96.2080	95.9353	196.0941	179.0193	387.6401	359.7747
20	94.8879	91.4134	191.8969	177.3781	388.6408	358.9294

**Table A4:** Raw rotational angle measurements before and after calibration for clockwise rotations on the asphalt surface.

Trial No.	90°		180°		360°	
	Before Calibration (°)	After Calibration (°)	Before Calibration (°)	After Calibration (°)	Before Calibration (°)	After Calibration (°)
1	100.6041	97.8776	196.0092	182.1615	385.361	357.7259
2	101.5195	96.5993	193.3399	177.8758	386.9687	357.4412
3	98.8460	91.6246	193.2726	181.8625	394.3669	358.0638
4	98.3007	95.1050	195.8206	184.3408	393.4593	357.0628
5	104.1998	94.5844	194.1045	173.6960	389.9714	357.1401
6	106.0035	96.2118	196.7471	177.8993	390.8987	357.7645
7	101.7300	90.4465	195.9884	182.0919	397.8982	357.6835
8	105.4983	94.4831	194.3938	179.3263	386.8758	359.6021
9	103.5560	95.6750	193.2806	181.3241	393.6271	351.9585
10	100.2609	88.5939	192.1951	178.9159	388.6021	359.4257
11	102.6086	90.0406	193.6282	180.3672	388.7228	359.3972
12	106.7416	87.8395	197.1969	179.5213	396.5104	355.2992
13	101.6261	92.2061	194.9461	183.6694	394.8803	357.1782
14	107.0730	94.2255	192.4759	181.0857	394.5481	357.5670
15	93.8173	96.9250	195.0328	181.5718	397.683	355.9849
16	104.7239	92.9222	195.4245	179.3504	389.8505	357.4441
17	101.8907	96.8224	193.6879	178.9894	394.4012	356.4271
18	101.0989	89.2449	195.0276	179.3110	387.7145	359.9505
19	102.3410	88.8088	195.4950	181.6231	392.1034	359.3030
20	96.2900	92.1900	193.0949	179.4910	388.7484	359.8438

**Table A5:** Raw rotational angle measurements before and after calibration for counterclockwise rotations on the ceramic tile surface.

Trial No.	90°		180°		360°	
	Before Calibration (°)	After Calibration (°)	Before Calibration (°)	After Calibration (°)	Before Calibration (°)	After Calibration (°)
1	95.8812	94.2292	193.3801	182.8637	387.3607	358.6828
2	100.9738	93.9305	192.2551	181.2339	389.4312	362.0294
3	99.5227	93.6318	195.0440	181.7743	388.7852	361.1729
4	101.3943	92.4141	195.4528	182.0856	390.0350	360.9479
5	100.1693	94.1364	194.8625	182.2394	383.9796	361.1769
6	100.8340	95.1035	187.0489	182.3966	389.7251	366.3713
7	100.1273	96.7432	196.4936	182.7584	394.4582	361.3954
8	97.5124	95.2654	194.3751	183.1210	389.1351	359.8433
9	98.6672	94.7722	203.4276	182.3415	390.0396	358.2891
10	100.3608	94.2791	195.8442	181.5644	388.5643	357.8507
11	101.6386	93.7326	195.8943	181.8318	395.2571	355.9902
12	101.1303	92.8270	196.4190	182.7146	383.8972	354.2663
13	101.1809	97.3710	195.7094	182.8877	401.9759	354.3127
14	100.5733	94.5357	196.9959	182.7152	402.6058	356.7025
15	102.3847	91.7004	196.0789	182.6964	383.6813	357.5584
16	105.5668	95.6485	197.2718	182.1268	388.7542	358.4211
17	99.9330	95.4448	202.7815	181.5554	383.1323	358.9864
18	100.6752	92.5487	196.9212	181.8984	394.7344	359.1398
19	101.1632	93.4547	195.6711	182.2416	390.0611	359.4037
20	101.0592	94.3608	195.8439	183.8622	389.3685	358.0999

**Table A6:** Raw rotational angle measurements before and after calibration for clockwise rotations on the ceramic tile surface.

Trial No.	90°		180°		360°	
	Before Calibration (°)	After Calibration (°)	Before Calibration (°)	After Calibration (°)	Before Calibration (°)	After Calibration (°)
1	90.2145	94.1853	195.8997	181.8665	388.6688	360.4851
2	100.6086	92.2236	197.0151	181.7581	389.6801	360.1264
3	101.4375	94.0202	194.2845	183.0453	388.8851	359.7904
4	99.8678	92.5193	195.4845	182.7684	388.8237	360.6522
5	102.3092	95.6731	193.1227	182.4950	387.5128	359.2284
6	101.3559	95.9378	203.3316	181.9745	386.4807	361.8276
7	99.0399	96.0767	203.1353	183.5148	386.4134	361.2739
8	100.0069	95.3440	203.1791	183.6960	385.5475	361.4866
9	103.1703	93.7108	195.0970	188.8787	399.1589	360.9242
10	99.4367	93.3867	195.7984	181.5832	378.3165	360.5524
11	100.1084	94.2394	195.9818	182.1843	382.6712	359.4183
12	101.5477	95.0920	202.4603	182.8945	384.8128	358.4620
13	95.4116	96.5881	197.5610	182.3738	382.4938	359.1307
14	100.7087	93.1260	195.7680	183.5947	389.1942	359.8674
15	103.4051	92.7515	197.9603	181.8256	377.9386	360.4573
16	98.1262	93.4263	208.7282	180.0168	388.5224	361.0238
17	100.5256	94.8818	196.4584	184.0231	376.6473	361.3852
18	101.0916	92.7486	210.2083	184.3623	382.5233	361.1248
19	101.6972	92.5728	196.2665	183.0636	376.4857	361.6018
20	99.8465	92.3970	200.1652	181.9384	388.5873	359.4827

## Appendix B. Raw Linear Displacement Calibration Data

**Table A7:** Raw travel distance measurements before and after longitudinal odometry calibration on the epoxy resin surface.

Trial No.	0.5		1.0		1.5	
	Before Calibration (m)	After Calibration (m)	Before Calibration (m)	After Calibration (m)	Before Calibration (m)	After Calibration (m)
1	0.469	0.491	0.935	1.008	1.424	1.531
2	0.471	0.495	0.938	1.000	1.422	1.520
3	0.452	0.506	0.946	1.013	1.426	1.514
4	0.467	0.501	0.945	1.012	1.419	1.509
5	0.461	0.492	0.944	1.005	1.428	1.516
6	0.467	0.503	0.944	1.016	1.426	1.505
7	0.464	0.494	0.955	1.026	1.439	1.527
8	0.477	0.494	1.024	1.006	1.425	1.497
9	0.452	0.493	0.951	1.002	1.432	1.523
10	0.467	0.494	0.950	1.002	1.433	1.505
11	0.460	0.507	0.943	1.010	1.426	1.518
12	0.465	0.496	0.948	0.996	1.423	1.518
13	0.468	0.508	0.949	1.013	1.422	1.515
14	0.464	0.495	0.953	1.002	1.422	1.511
15	0.467	0.491	0.953	1.001	1.425	1.522
16	0.465	0.498	0.943	1.013	1.421	1.506
17	0.467	0.498	0.946	1.001	1.427	1.504
18	0.461	0.495	0.944	1.000	1.414	1.508
19	0.466	0.492	0.958	1.009	1.428	1.517
20	0.470	0.495	0.938	1.009	1.428	1.514
21	0.475	0.495	0.943	1.028	1.429	1.512
22	0.466	0.497	0.945	1.011	1.419	1.534
23	0.466	0.496	0.938	0.996	1.438	1.513
24	0.470	0.495	0.958	0.999	1.418	1.509
25	0.464	0.493	0.942	1.002	1.428	1.512
26	0.466	0.507	0.951	1.019	1.422	1.511
27	0.467	0.485	0.954	0.983	1.429	1.514
28	0.475	0.491	0.951	0.997	1.424	1.515
29	0.466	0.508	0.937	1.005	1.428	1.525
30	0.469	0.492	0.953	1.000	1.429	1.510

**Table A8:** Raw travel distance measurements before and after longitudinal odometry calibration on the asphalt surface.

Trial No.	0.5		1.0		1.5	
	Before Calibration (m)	After Calibration (m)	Before Calibration (m)	After Calibration (m)	Before Calibration (m)	After Calibration (m)
1	0.469	0.499	0.942	1.001	1.421	1.521
2	0.461	0.495	0.946	1.004	1.423	1.507
3	0.463	0.503	0.957	0.996	1.434	1.526
4	0.468	0.506	0.952	0.995	1.429	1.503
5	0.465	0.495	0.939	1.012	1.426	1.519
6	0.476	0.495	0.945	1.017	1.426	1.532
7	0.468	0.506	0.944	1.005	1.433	1.506
8	0.465	0.501	0.943	1.014	1.423	1.512
9	0.461	0.493	0.958	1.009	1.429	1.515
10	0.468	0.502	0.949	1.007	1.425	1.513
11	0.463	0.493	0.939	1.009	1.425	1.502
12	0.473	0.493	0.953	1.019	1.432	1.515
13	0.467	0.498	0.966	1.005	1.435	1.506
14	0.459	0.484	0.953	1.019	1.437	1.518
15	0.467	0.485	0.937	0.983	1.433	1.507
16	0.468	0.493	0.944	1.013	1.426	1.527
17	0.461	0.497	0.955	1.006	1.439	1.508
18	0.467	0.498	0.942	1.003	1.424	1.512
19	0.466	0.491	0.955	1.006	1.428	1.521
20	0.469	0.487	0.952	0.988	1.425	1.504
21	0.468	0.505	0.941	1.004	1.426	1.516
22	0.467	0.495	0.946	1.009	1.429	1.525
23	0.472	0.497	0.926	1.018	1.421	1.501
24	0.471	0.487	0.947	1.001	1.437	1.516
25	0.459	0.493	0.945	0.998	1.428	1.517
26	0.461	0.497	0.939	1.001	1.419	1.521
27	0.469	0.489	0.957	1.013	1.432	1.504
28	0.469	0.499	0.938	1.008	1.439	1.504
29	0.469	0.492	0.944	1.001	1.429	1.519
30	0.486	0.502	0.948	1.012	1.429	1.537

**Table A9:** Raw travel distance measurements before and after longitudinal odometry calibration on the ceramic tile surface.

Trial No.	0.5		1.0		1.5	
	Before Calibration (m)	After Calibration (m)	Before Calibration (m)	After Calibration (m)	Before Calibration (m)	After Calibration (m)
1	0.469	0.494	0.96	1.01	1.426	1.517
2	0.472	0.492	0.94	1.006	1.421	1.517
3	0.471	0.508	0.95	1.014	1.426	1.517
4	0.469	0.496	0.95	0.999	1.422	1.509
5	0.464	0.497	0.94	1.003	1.431	1.516
6	0.473	0.501	0.95	1.002	1.425	1.517
7	0.462	0.489	0.95	0.993	1.421	1.509
8	0.465	0.498	0.94	1.008	1.424	1.53
9	0.463	0.484	0.95	1.008	1.428	1.518
10	0.466	0.488	0.94	1.005	1.423	1.505
11	0.478	0.497	0.95	1.003	1.421	1.52
12	0.456	0.501	0.95	0.993	1.427	1.507
13	0.469	0.497	0.96	1.002	1.427	1.521
14	0.457	0.496	0.94	1.002	1.423	1.524
15	0.463	0.494	0.96	0.999	1.423	1.508
16	0.472	0.487	0.93	1.004	1.427	1.522
17	0.466	0.492	0.95	1.009	1.418	1.518
18	0.467	0.493	0.95	1.022	1.418	1.521
19	0.462	0.503	0.95	1.007	1.422	1.53
20	0.469	0.498	0.94	1.008	1.425	1.512
21	0.462	0.485	0.95	1.005	1.427	1.508
22	0.467	0.498	0.94	0.989	1.434	1.507
23	0.466	0.494	0.94	1.005	1.433	1.508
24	0.462	0.492	0.95	1.006	1.425	1.514
25	0.477	0.502	0.95	1.027	1.426	1.517
26	0.469	0.503	0.94	1.004	1.427	1.517
27	0.455	0.502	0.95	1.008	1.426	1.521
28	0.467	0.491	0.95	1.005	1.424	1.515
29	0.462	0.494	0.95	0.995	1.428	1.526
30	0.473	0.498	0.95	1.015	1.421	1.512

## Appendix C. Detailed Results of the ZUPT-Based Pose Estimation Experiments

### Appendix C.1. Dataset-Level/Odom-Referenced Positional Deviation Metrics

**Table A10:** Dataset-level positional error metrics for the EKF under fixed-covariance and ZUPT-driven adaptive-covariance configurations.

Surface Type	Method	Dataset No.	Position RMSE (m)	Position MAE (m)	Maximum Position Error (m)	Standard Deviation of Position Error (m)
Epoxy resin surface	Fixed covariance	1	0.44181248	0.364323449	0.792976814	0.249933375
Epoxy resin surface	Fixed covariance	2	0.258455162	0.257466959	0.298152277	0.022579544
Epoxy resin surface	Fixed covariance	3	0.42926525	0.344373962	0.810593461	0.256271787
Epoxy resin surface	Fixed covariance	4	0.153970011	0.136880981	0.2431089	0.070500789
Epoxy resin surface	Fixed covariance	5	0.414113377	0.390415696	0.625805156	0.138077779
Epoxy resin surface	Fixed covariance	6	0.464467414	0.380846742	0.851804805	0.265867894
Epoxy resin surface	ZUPT	1	0.168707657	0.163473389	0.269154303	0.041698016
Epoxy resin surface	ZUPT	2	0.215444017	0.20191701	0.361466467	0.075137511
Epoxy resin surface	ZUPT	3	0.127110509	0.121700478	0.181366946	0.036688895
Epoxy resin surface	ZUPT	4	0.181343394	0.169014119	0.256614159	0.065724076
Epoxy resin surface	ZUPT	5	0.113447181	0.11180019	0.1461086	0.019260856
Epoxy resin surface	ZUPT	6	0.152467341	0.149709034	0.226376314	0.028870318
Asphalt surface	Fixed covariance	1	0.542143216	0.440691996	1.300058458	0.315768636
Asphalt surface	Fixed covariance	2	0.310698522	0.292650605	0.496299979	0.104351304
Asphalt surface	Fixed covariance	3	0.419384061	0.358394929	0.835415554	0.217798222
Asphalt surface	Fixed covariance	4	0.338816446	0.317808952	0.488422867	0.117448089
Asphalt surface	Fixed covariance	5	0.414996351	0.366726355	0.785828617	0.194251774
Asphalt surface	Fixed covariance	6	0.426862059	0.413066615	0.576611939	0.107643807
Asphalt surface	ZUPT	1	0.28137459	0.262918565	0.514162203	0.100227182
Asphalt surface	ZUPT	2	0.27482522	0.264836048	0.456406824	0.073421857
Asphalt surface	ZUPT	3	0.174734424	0.167278936	0.384697005	0.050496301
Asphalt surface	ZUPT	4	0.19119689	0.179487797	0.325423265	0.065881569
Asphalt surface	ZUPT	5	0.237788196	0.232378658	0.399679074	0.050431985
Asphalt surface	ZUPT	6	0.263700879	0.247196383	0.461266704	0.091826476
Ceramic tile surface	Fixed covariance	1	0.223726	0.200821	0.372997	0.098612
Ceramic tile surface	Fixed covariance	2	0.082036	0.058509	0.247031	0.057503
Ceramic tile surface	Fixed covariance	3	0.039951	0.03513	0.103653	0.019027
Ceramic tile surface	Fixed covariance	4	0.054366	0.046265	0.146265	0.028552
Ceramic tile surface	Fixed covariance	5	0.038818	0.033972	0.079256	0.018781
Ceramic tile surface	Fixed covariance	6	0.046447	0.044238	0.067113	0.014153
Ceramic tile surface	ZUPT	1	0.036442	0.031411	0.082246	0.018477
Ceramic tile surface	ZUPT	2	0.03634	0.030926	0.084393	0.019084
Ceramic tile surface	ZUPT	3	0.026095	0.023423	0.05791	0.011503
Ceramic tile surface	ZUPT	4	0.034849	0.031719	0.067651	0.014435
Ceramic tile surface	ZUPT	5	0.036749	0.034384	0.061931	0.01297
Ceramic tile surface	ZUPT	6	0.038182	0.035645	0.063068	0.013687

**Table A11:** Dataset-level positional error metrics for the UKF under fixed-covariance and ZUPT-driven adaptive-covariance configurations.

Surface Type	Method	Dataset No.	Position RMSE (m)	Position MAE (m)	Maximum Position Error (m)	Standard Deviation of Position Error (m)
Epoxy resin surface	Fixed covariance	1	0.414468042	0.338399047	0.743974747	0.239311183
Epoxy resin surface	Fixed covariance	2	0.157157607	0.149862642	0.227850582	0.047325488
Epoxy resin surface	Fixed covariance	3	0.355737296	0.336139281	0.561491282	0.116444869
Epoxy resin surface	Fixed covariance	4	0.251371208	0.24911519	0.3370462	0.033602175
Epoxy resin surface	Fixed covariance	5	0.174854294	0.168690953	0.289194921	0.046015067
Epoxy resin surface	Fixed covariance	6	0.536412323	0.433693436	1.27875977	0.315671007
Epoxy resin surface	ZUPT	1	0.171540593	0.163659156	0.289355335	0.051398984
Epoxy resin surface	ZUPT	2	0.165952668	0.160546365	0.230893066	0.042013721
Epoxy resin surface	ZUPT	3	0.105318295	0.098524563	0.162240293	0.037213624
Epoxy resin surface	ZUPT	4	0.237677789	0.236939721	0.27414816	0.018716311
Epoxy resin surface	ZUPT	5	0.200865737	0.199965628	0.235989123	0.018994523
Epoxy resin surface	ZUPT	6	0.139818102	0.13678595	0.215264447	0.028960414
Asphalt surface	Fixed covariance	1	0.536412323	0.433693436	1.27875977	0.315671007
Asphalt surface	Fixed covariance	2	0.282352342	0.269125572	0.406040087	0.08540651
Asphalt surface	Fixed covariance	3	0.363451147	0.333184051	0.551759297	0.145207179
Asphalt surface	Fixed covariance	4	0.393915338	0.384420357	0.491707538	0.085966755
Asphalt surface	Fixed covariance	5	0.400012239	0.37280502	0.697329808	0.145004166
Asphalt surface	Fixed covariance	6	0.392027995	0.380649138	0.528823694	0.09376664
Asphalt surface	ZUPT	1	0.215573127	0.212653696	0.337543392	0.035357866
Asphalt surface	ZUPT	2	0.260185476	0.252969843	0.388132917	0.060850148
Asphalt surface	ZUPT	3	0.240982961	0.236059562	0.356033243	0.048463089
Asphalt surface	ZUPT	4	0.183701664	0.181567776	0.291176848	0.027918528
Asphalt surface	ZUPT	5	0.196692927	0.194812542	0.268389449	0.027132655
Asphalt surface	ZUPT	6	0.238104292	0.224313633	0.397172293	0.079856422
Ceramic tile surface	Fixed covariance	1	0.086625	0.071969	0.194961	0.048213
Ceramic tile surface	Fixed covariance	2	0.071443	0.066008	0.228245	0.027332
Ceramic tile surface	Fixed covariance	3	0.054366	0.046265	0.146265	0.028552
Ceramic tile surface	Fixed covariance	4	0.039997	0.035729	0.08121	0.01798
Ceramic tile surface	Fixed covariance	5	0.042443	0.039757	0.069069	0.01486
Ceramic tile surface	Fixed covariance	6	0.045964	0.040121	0.105888	0.022427
Ceramic tile surface	ZUPT	1	0.033102	0.029666	0.069988	0.014685
Ceramic tile surface	ZUPT	2	0.031802	0.028696	0.067205	0.013707
Ceramic tile surface	ZUPT	3	0.034849	0.031719	0.067651	0.014435
Ceramic tile surface	ZUPT	4	0.033641	0.030461	0.073928	0.014277
Ceramic tile surface	ZUPT	5	0.030629	0.025986	0.080121	0.016214
Ceramic tile surface	ZUPT	6	0.026459	0.023579	0.055738	0.012004

**Table A12:** Dataset-level positional error metrics for the RCKF under fixed-covariance and ZUPT-driven adaptive-covariance configurations.

Surface Type	Method	Dataset No.	Position RMSE (m)	Position MAE (m)	Maximum Position Error (m)	Standard Deviation of Position Error (m)
Epoxy resin surface	Fixed covariance	1	0.215613961	0.192750712	0.365874611	0.09662579
Epoxy resin surface	Fixed covariance	2	0.299683289	0.290703034	0.454254821	0.072813594
Epoxy resin surface	Fixed covariance	3	0.242259318	0.240714189	0.322621391	0.02731769
Epoxy resin surface	Fixed covariance	4	0.269995413	0.266730029	0.36204277	0.041864242
Epoxy resin surface	Fixed covariance	5	0.263780083	0.26030617	0.357497556	0.04266884
Epoxy resin surface	Fixed covariance	6	0.133749239	0.131920062	0.171647466	0.022044414
Epoxy resin surface	ZUPT	1	0.097330738	0.084026061	0.16287596	0.049121214
Epoxy resin surface	ZUPT	2	0.240550716	0.239730444	0.282272216	0.019848454
Epoxy resin surface	ZUPT	3	0.17966157	0.174147264	0.242581744	0.044170241
Epoxy resin surface	ZUPT	4	0.097968171	0.095849379	0.13743511	0.020264727
Epoxy resin surface	ZUPT	5	0.194451841	0.193480351	0.224195865	0.019413193
Epoxy resin surface	ZUPT	6	0.139237464	0.136413792	0.211862195	0.0278989
Asphalt surface	Fixed covariance	1	0.336276544	0.293770416	0.658483024	0.163648576
Asphalt surface	Fixed covariance	2	0.160382471	0.15837084	0.236307877	0.025322199
Asphalt surface	Fixed covariance	3	0.264531196	0.222716246	0.529968089	0.142738318
Asphalt surface	Fixed covariance	4	0.230342232	0.224836102	0.323991572	0.050062671
Asphalt surface	Fixed covariance	5	0.319063547	0.26210354	0.745556884	0.181943071
Asphalt surface	Fixed covariance	6	0.320684399	0.314041676	0.441116955	0.064933114
Asphalt surface	ZUPT	1	0.234732096	0.209238971	0.445665432	0.106387075
Asphalt surface	ZUPT	2	0.291886294	0.281204971	0.483689403	0.078239205
Asphalt surface	ZUPT	3	0.198154937	0.195302419	0.342280334	0.033501406
Asphalt surface	ZUPT	4	0.157315863	0.148989089	0.292047641	0.050502792
Asphalt surface	ZUPT	5	0.197731565	0.195867007	0.265062709	0.02709035
Asphalt surface	ZUPT	6	0.235329371	0.220959277	0.395918349	0.080974755
Ceramic tile surface	Fixed covariance	1	0.047331	0.043443	0.078994	0.018786
Ceramic tile surface	Fixed covariance	2	0.040896	0.036297	0.088938	0.018843
Ceramic tile surface	Fixed covariance	3	0.049889	0.04133	0.150081	0.027941
Ceramic tile surface	Fixed covariance	4	0.411032	0.353979	0.775643	0.208916
Ceramic tile surface	Fixed covariance	5	0.03977	0.035905	0.083742	0.017102
Ceramic tile surface	Fixed covariance	6	0.03634	0.030926	0.084393	0.019084
Ceramic tile surface	ZUPT	1	0.034886	0.031574	0.06633	0.014835
Ceramic tile surface	ZUPT	2	0.033894	0.029618	0.076565	0.016479
Ceramic tile surface	ZUPT	3	0.033994	0.030162	0.07087	0.01568
Ceramic tile surface	ZUPT	4	0.033155	0.02979	0.059282	0.014556
Ceramic tile surface	ZUPT	5	0.035063	0.033141	0.054805	0.011447
Ceramic tile surface	ZUPT	6	0.032614	0.030226	0.055129	0.01225

**Table A13:** Dataset-level positional error metrics for the graph-based SLAM optimization framework under fixed-covariance and ZUPT-driven adaptive-covariance configurations.

Surface Type	Method	Dataset No.	Position RMSE (m)	Position MAE (m)	Maximum Position Error (m)	Standard Deviation of Position Error (m)
Epoxy resin surface	Fixed covariance	1	0.044007	0.040231	0.067624	0.017835
Epoxy resin surface	Fixed covariance	2	0.034439	0.030517	0.057638	0.01596
Epoxy resin surface	Fixed covariance	3	0.03801	0.032673	0.088348	0.019424
Epoxy resin surface	Fixed covariance	4	0.037668	0.033992	0.062511	0.01623
Epoxy resin surface	Fixed covariance	5	0.039984	0.035838	0.104484	0.017731
Epoxy resin surface	Fixed covariance	6	0.035415	0.032983	0.05658	0.012899
Epoxy resin surface	ZUPT	1	0.025008	0.022833	0.049857	0.010199
Epoxy resin surface	ZUPT	2	0.027682	0.024359	0.046519	0.01315
Epoxy resin surface	ZUPT	3	0.030698	0.027685	0.060172	0.013263
Epoxy resin surface	ZUPT	4	0.036842	0.032207	0.083125	0.017889
Epoxy resin surface	ZUPT	5	0.023411	0.021198	0.045746	0.009937
Epoxy resin surface	ZUPT	6	0.040061	0.037246	0.070308	0.014751
Asphalt surface	Fixed covariance	1	0.482302	0.407299	1.243478	0.258307
Asphalt surface	Fixed covariance	2	0.05728	0.040939	0.245807	0.040062
Asphalt surface	Fixed covariance	3	0.052699	0.047971	0.112096	0.021817
Asphalt surface	Fixed covariance	4	0.086164	0.070984	0.193148	0.048842
Asphalt surface	Fixed covariance	5	0.039697	0.034368	0.097574	0.019867
Asphalt surface	Fixed covariance	6	0.04449	0.038282	0.095706	0.022669
Asphalt surface	ZUPT	1	0.087717	0.071604	0.260883	0.050666
Asphalt surface	ZUPT	2	0.035766	0.032407	0.085065	0.015132
Asphalt surface	ZUPT	3	0.048105	0.043311	0.09212	0.020934
Asphalt surface	ZUPT	4	0.066057	0.057214	0.148549	0.033016
Asphalt surface	ZUPT	5	0.054958	0.047754	0.14264	0.027201
Asphalt surface	ZUPT	6	0.060727	0.052974	0.167896	0.029689
Ceramic tile surface	Fixed covariance	1	0.068357	0.059151	0.16833	0.034261
Ceramic tile surface	Fixed covariance	2	0.040702	0.038249	0.059657	0.013916
Ceramic tile surface	Fixed covariance	3	0.081717	0.079407	0.113443	0.019292
Ceramic tile surface	Fixed covariance	4	0.043837	0.040575	0.069724	0.016594
Ceramic tile surface	Fixed covariance	5	0.039779	0.03446	0.073869	0.019871
Ceramic tile surface	Fixed covariance	6	0.039555	0.037605	0.058004	0.012265
Ceramic tile surface	ZUPT	1	0.036547	0.031972	0.097233	0.017707
Ceramic tile surface	ZUPT	2	0.030073	0.026156	0.085824	0.014841
Ceramic tile surface	ZUPT	3	0.03434	0.029809	0.079134	0.017049
Ceramic tile surface	ZUPT	4	0.032264	0.028743	0.058419	0.014657
Ceramic tile surface	ZUPT	5	0.029284	0.0263	0.062332	0.012878
Ceramic tile surface	ZUPT	6	0.03172	0.028602	0.063979	0.013716
